# Supplementary material for: Hydrolase mimic via second coordination sphere engineering in metal-organic frameworks for environmental remediation
Source: Nat Commun. 2023 Sep 25;14:5974. doi: 10.1038/s41467-023-41716-6 (PMC10520056; doi:10.1038/s41467-023-41716-6)
Supplement: Supplementary file 1 — Supplementary Information [file 41467_2023_41716_MOESM1_ESM.pdf]

Supplementary Information

Hydrolase mimic via second coordination sphere engineering in metal-organic frameworks for environmental remediation

Xin Yuan[1,4], Xiaoling Wu[1,4*], Jun Xiong[1], Binhang Yan[2], Ruichen Gao[1], Shuli Liu[1], Minhua Zong[1], Jun Ge[3*] and Wenyong Lou[1*]

[1]Lab of Applied Biocatalysis, School of Food Science and Technology, South China University of Technology, Guangzhou 510640, Guangdong, China.

[2]Department of Chemical Engineering, Tsinghua University, Beijing 100084, China

[3]Key Laboratory of Industrial Biocatalysis, Ministry of Education, Department of Chemical Engineering, Tsinghua University, Beijing 100084, China

[4]These authors contributed equally: Xin Yuan, Xiaoling Wu

**Email: wuxl18@scut.edu.cn; junge@tsinghua.edu.cn; wylou@scut.edu.cn*

Supplementary Figures

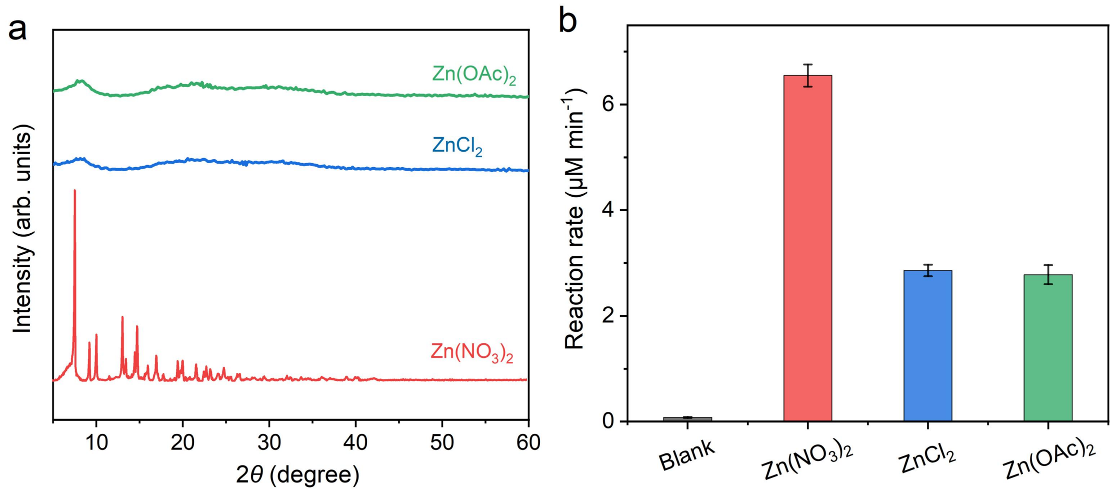

Supplementary Fig. 1. (a) The PXRD patterns of ZAF(Ser) synthesized with different zinc sources and (b) the corresponding reaction rate towards hydrolysis of hippuryl-L-phenylalanine (HPPA). Data were represented as mean ± SD (n = 3). Source data are provided as a Source Data file.

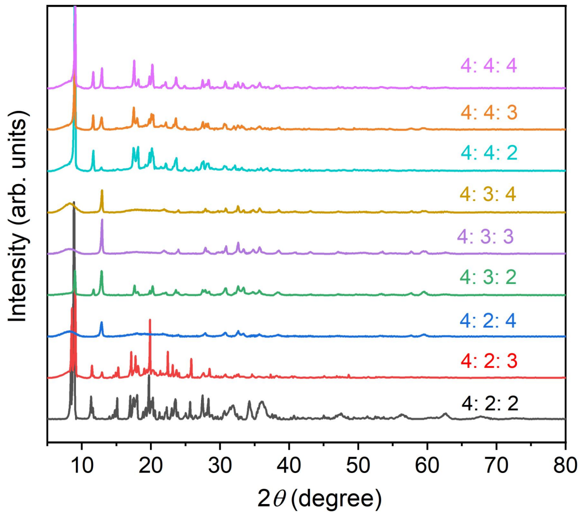

Supplementary Fig. 2. PXRD patterns of ZAF(Ser) synthesized by varying the molar ratios of Zn: BTA: Ser. Source data are provided as a Source Data file.

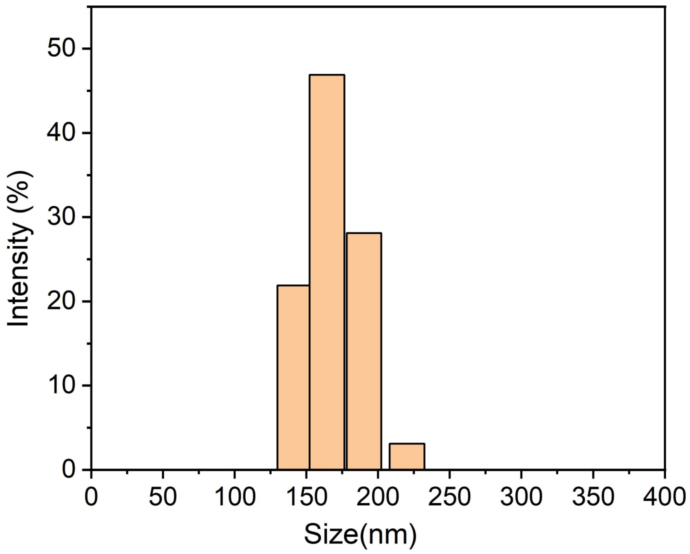

Supplementary Fig. 3. Size distribution of ZAF(Ser) determined by DLS. Source data are provided as a Source Data file.

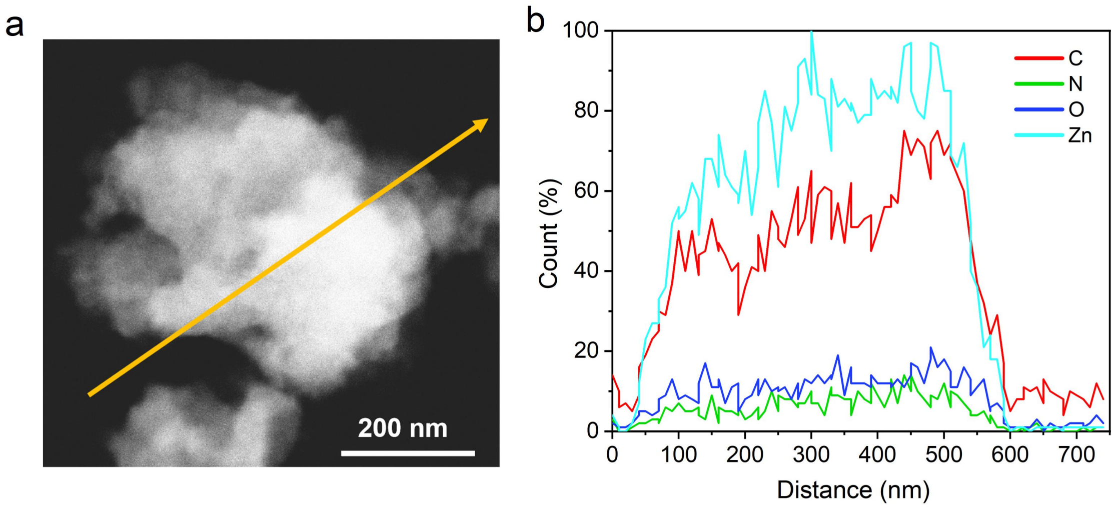

Supplementary Fig. 4. (a) HAADF-STEM image and (b) corresponding EDS line-scan elemental distribution curves of C, N, O and Zn for ZAF(Ser). Source data are provided as a Source Data file.

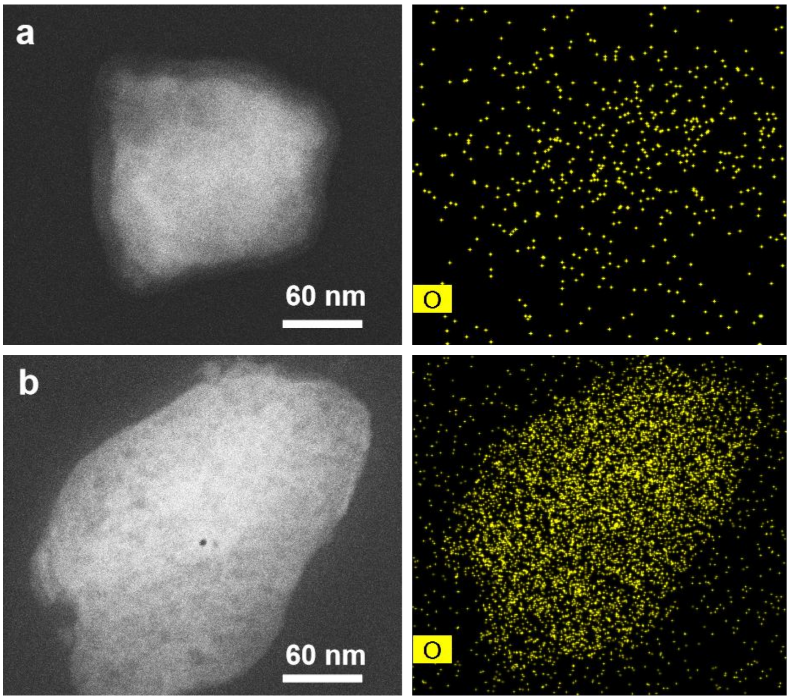

Supplementary Fig. 5. HAADF-STEM images of (a) ZAF and (b) ZAF(Ser) and the corresponding EDS elemental mapping images of O with prolonged scanning time 240 s.

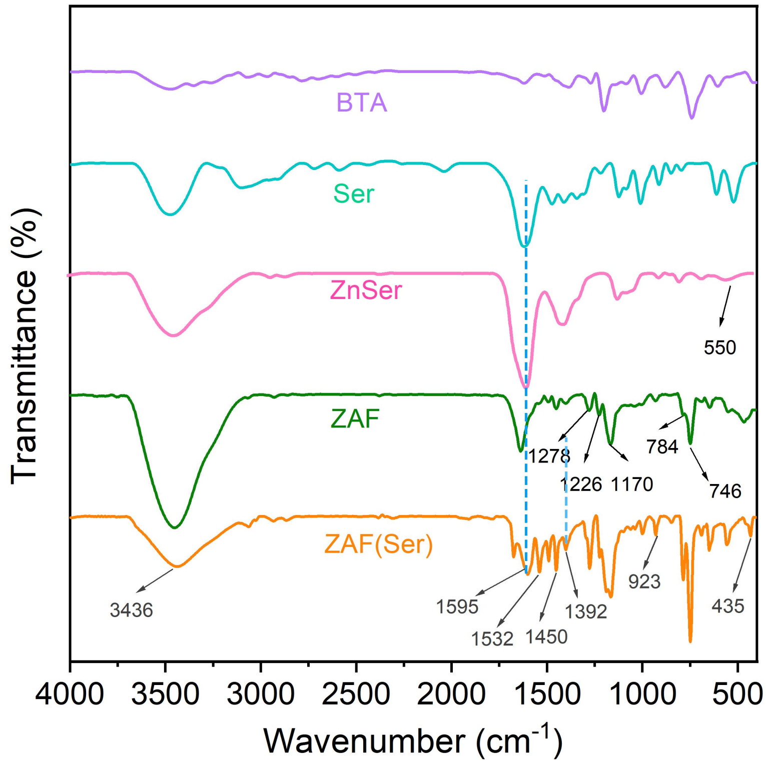

Supplementary Fig. 6. FT-IR spectra of ZAF(Ser), ZAF, Zn(Ser), Ser and BTA, respectively. Source data are provided as a Source Data file.

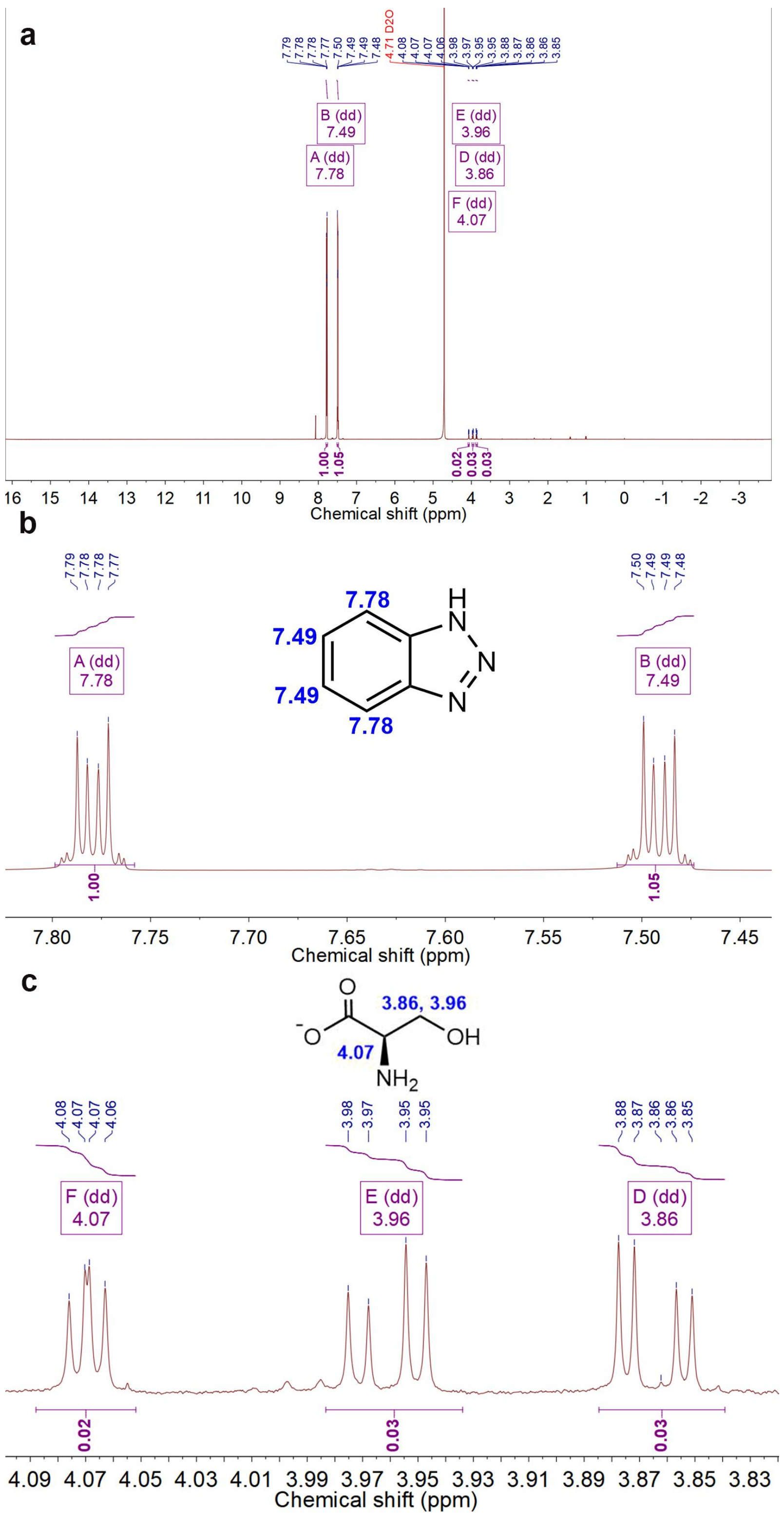

Supplementary Fig. 7. (a) ^{1}H NMR spectra of digested ZAF(Ser). (b) Partial enlarged ^{1}H NMR spectra of benzotriazole (BTA). (c) Partial enlarged ^{1}H NMR spectrum of serine (Ser).

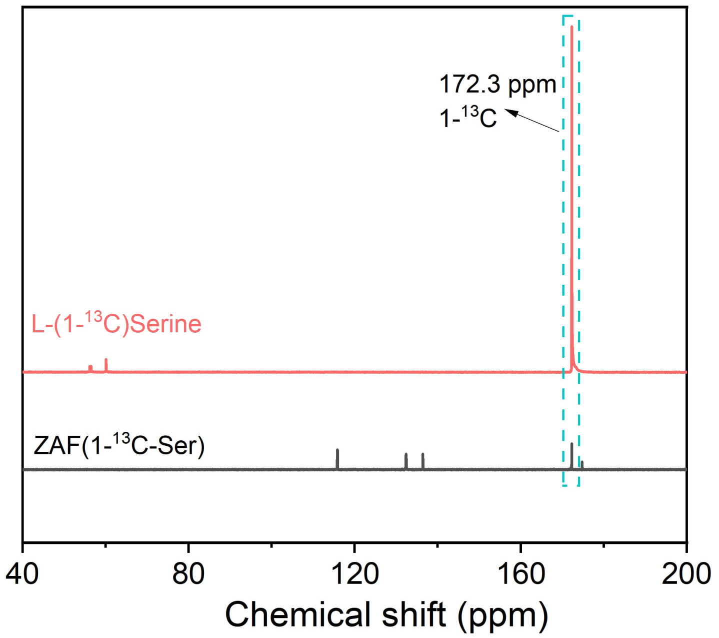

Supplementary Fig. 8. ^{13}C NMR spectra for ZAF(Ser) with 1-^{13}C isotopically labeled L-serine (1-^{13}C-Ser). Source data are provided as a Source Data file.

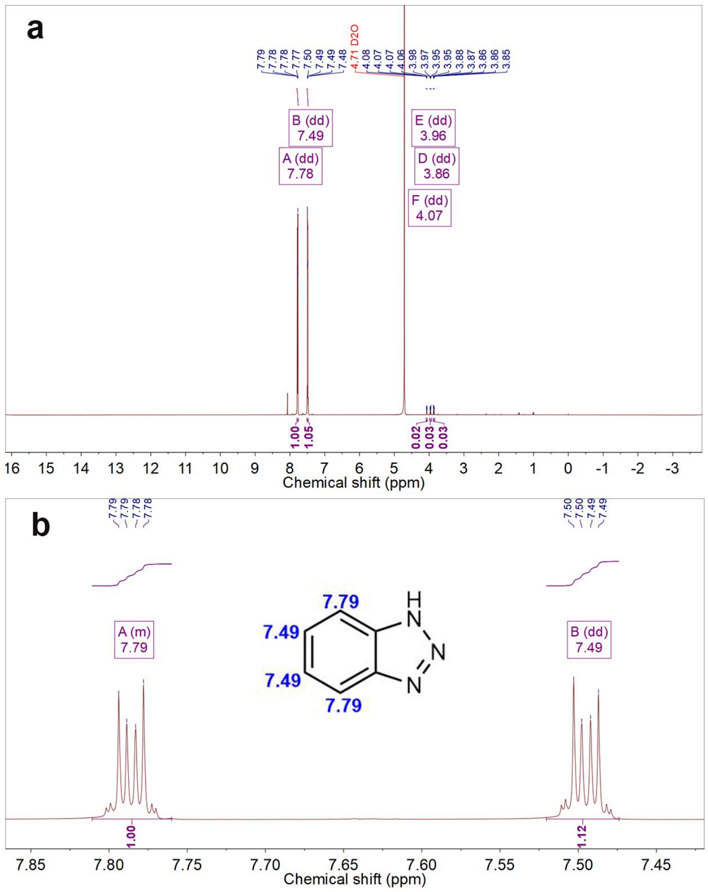

Supplementary Fig. 9. ^{1}H NMR spectra of digested ZAF. (b) Partial enlarged ^{1}H NMR spectra of benzotriazole (BTA).

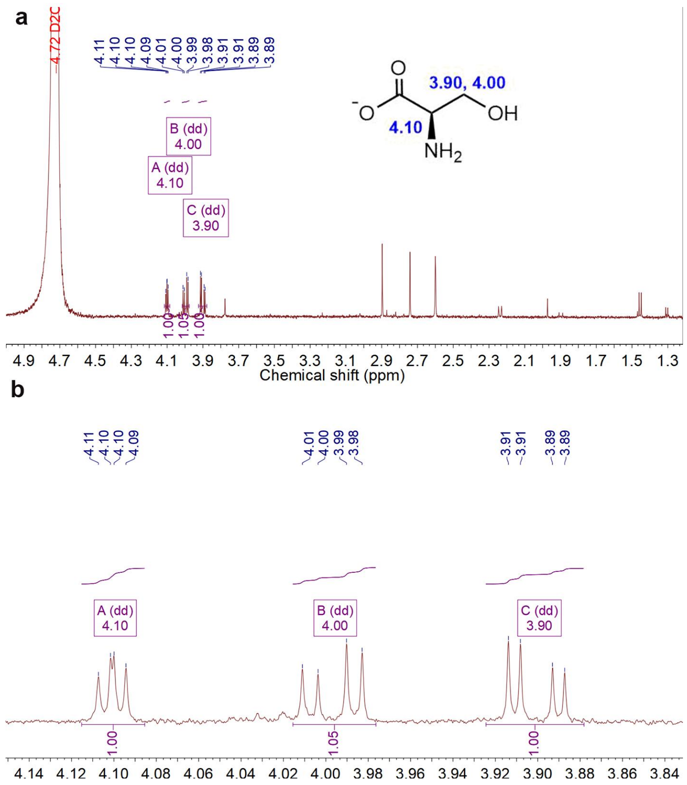

Supplementary Fig. 10. (a) ^{1}H NMR spectrum of digested ZnSer; (b) Partial enlarged ^{1}H NMR spectrum of serine (Ser).

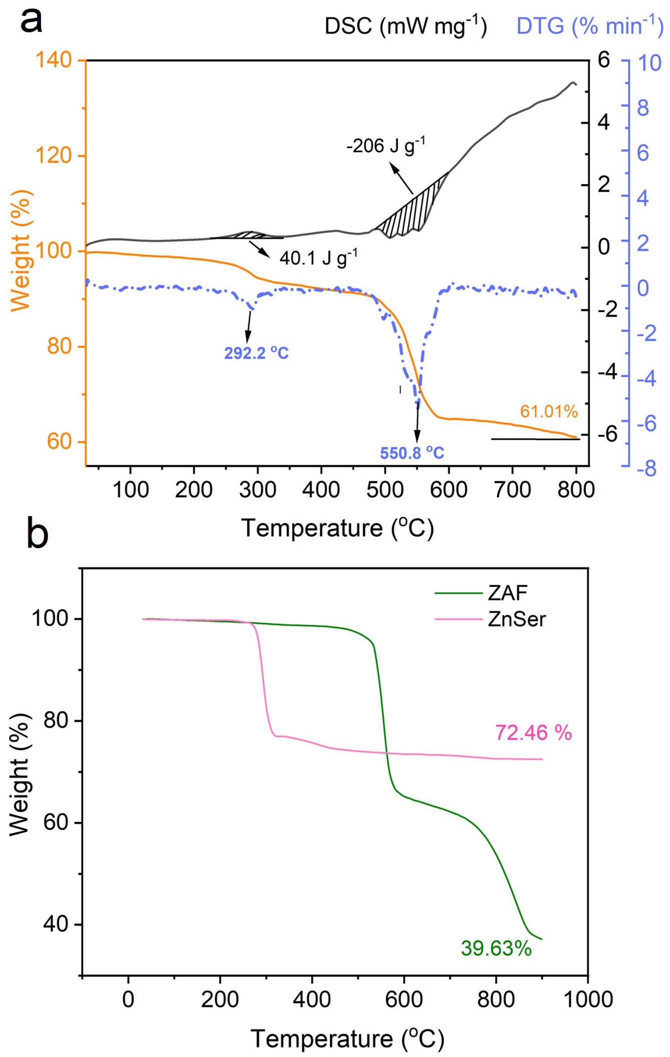

Supplementary Fig. 11. (a) TGA and DSC analysis of ZAF(Ser). (b) TGA analysis of ZAF and ZnSer. Source data are provided as a Source Data file.

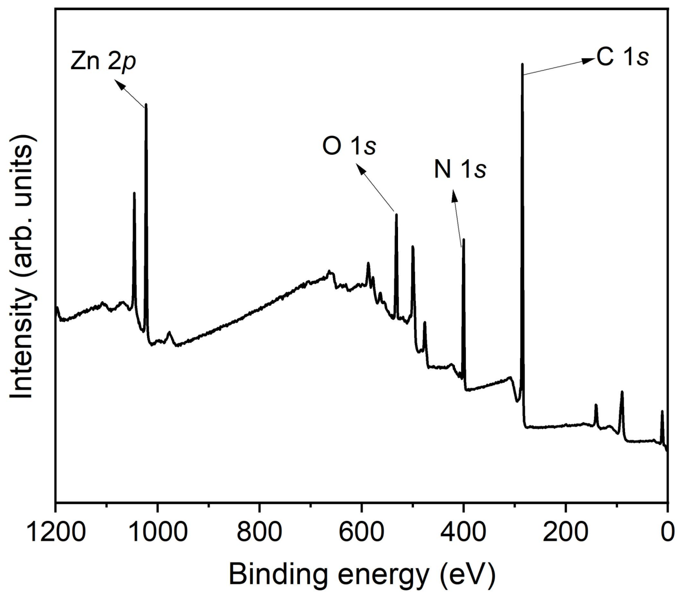

Supplementary Fig. 12. The survey XPS spectrum of ZAF(Ser). Source data are provided as a Source Data file.

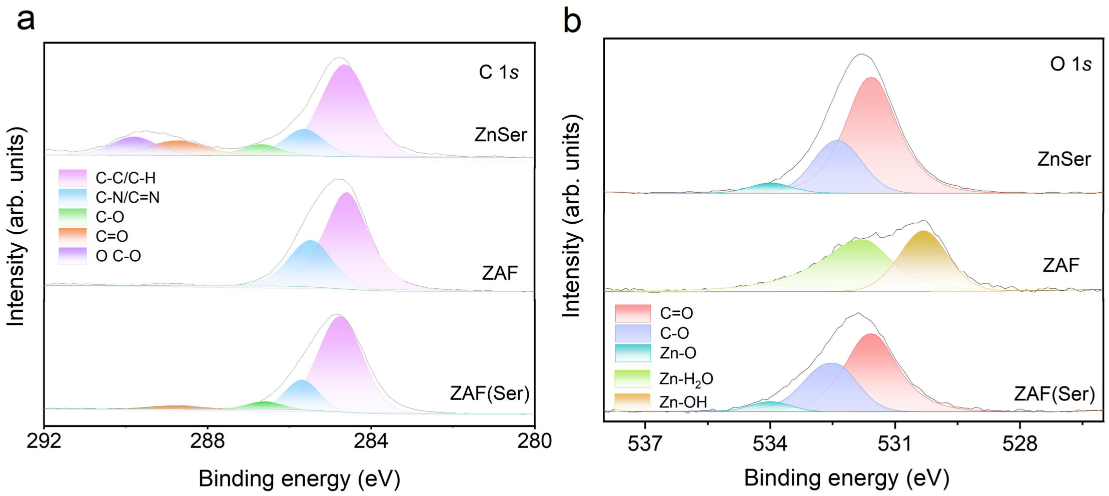

Supplementary Fig. 13. High-resolution spectra of C 1*s* (a) and O 1*s* (b) of ZAF(Ser), ZAF and ZnSer, respectively. Source data are provided as a Source Data file.

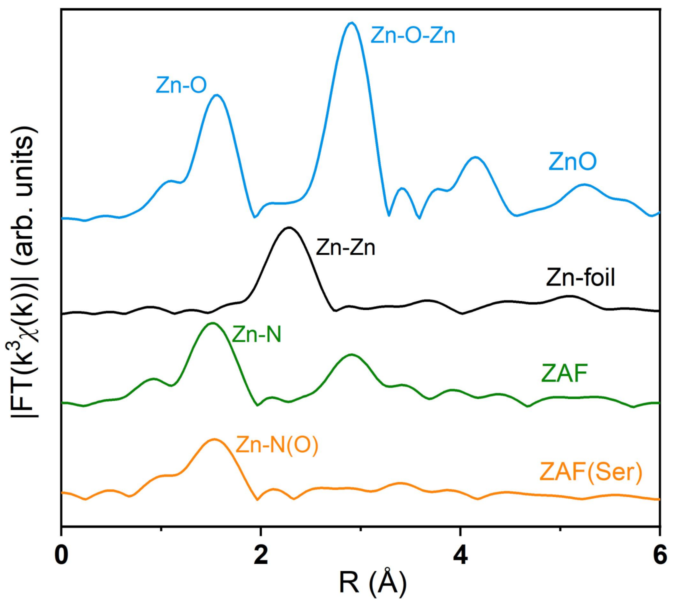

Supplementary Fig. 14. Fourier transforms of k^3-weighted Zn *K*-edge EXAFS of ZAF(Ser), ZAF, ZnO and Zn-foil, respectively. Source data are provided as a Source Data file.

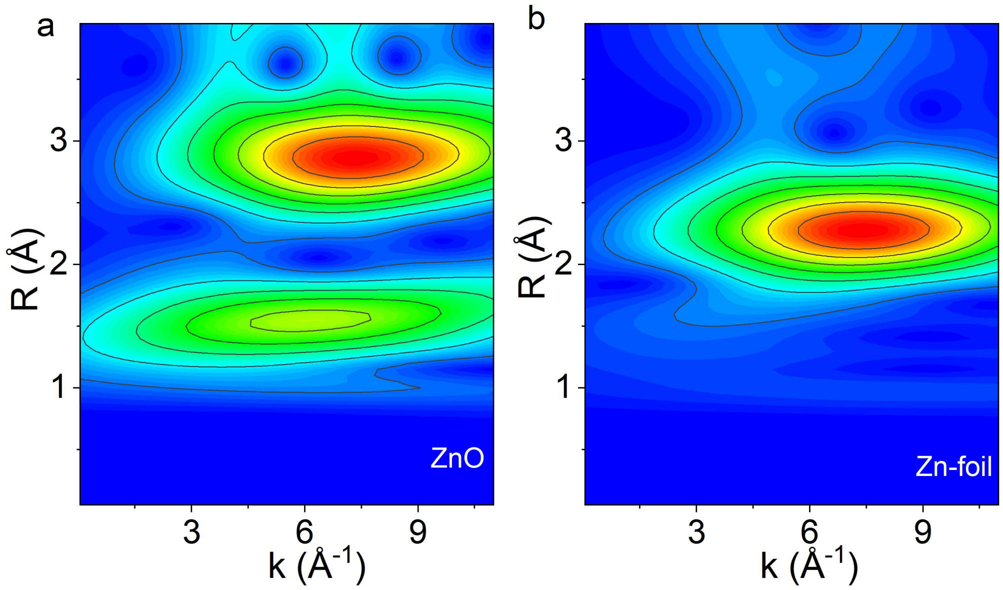

Supplementary Fig. 15. Wavelet transforms for the k^3-weighted Zn *K*-edge of EXAFS signals of ZnO (a) and Zn foil (b). Source data are provided as a Source Data file.

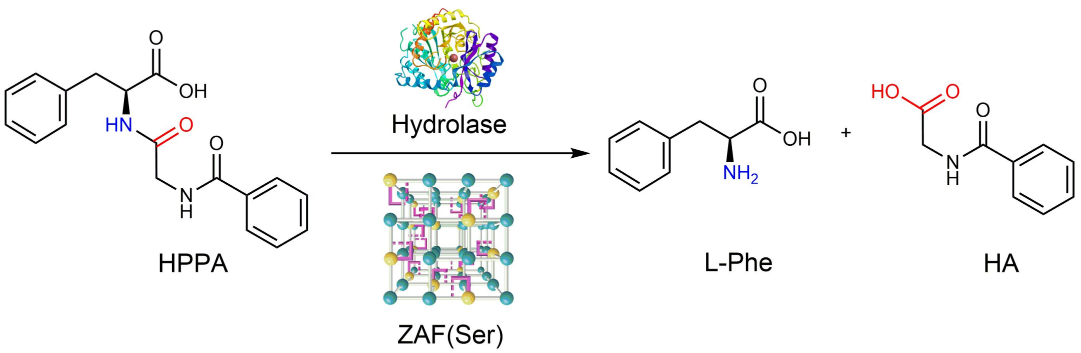

Supplementary Fig. 16. Hydrolysis of hippuryl-L-phenylalanine (HPPA) catalyzed by natural enzyme or ZAF(Ser) to generate L-phenylalanine (L-Phe) and hippuric acid (HA), concentration of which is determined by HPLC.

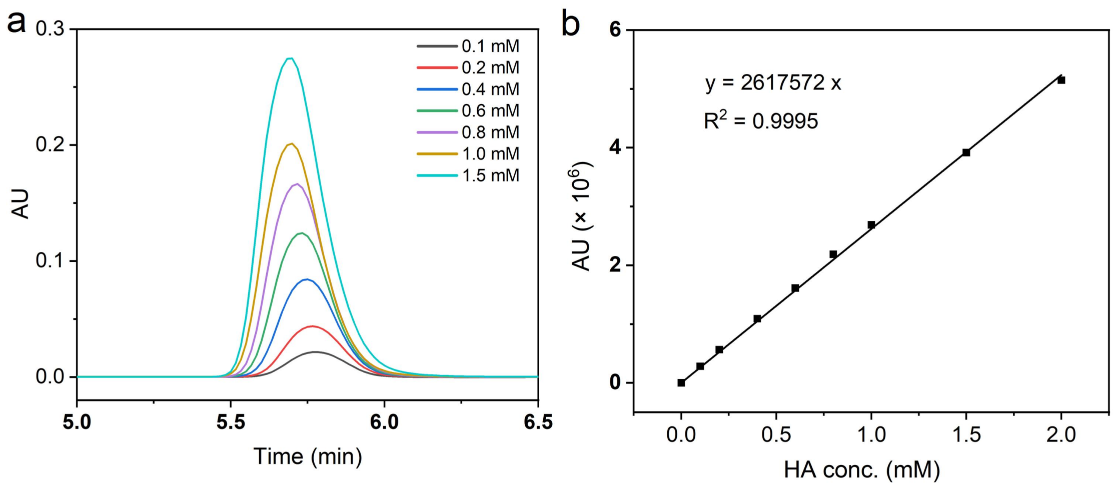

Supplementary Fig. 17. Quantitative assays of the hippuric acid (HA). (a) The HPLC diagram of HA with different concentrations; (b) The standard curve of HA with good linear relationship. Source data are provided as a Source Data file.

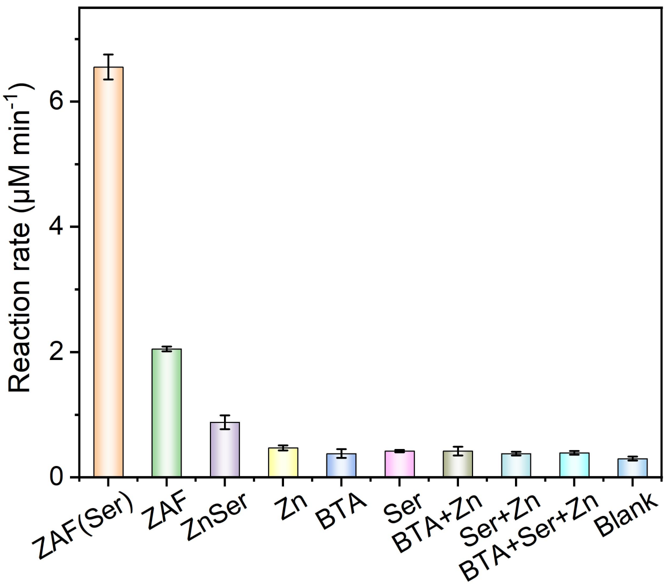

Supplementary Fig. 18. Reaction rate of ZAF(Ser), ZAF, ZnSer, Zn, BTA, Ser and physically mixture of the building blocks under identical assay conditions. Data were represented as mean ± SD (n = 3). The “BTA+Zn” represents the physical mixing of benzotriazole (BTA) and Zn ions. The “Ser+Zn” represents the physical mixing of serine (Ser) and Zn ions. The “BTA+Ser+Zn” represents the physical mixing of BTA, Ser and Zn ions. Source data are provided as a Source Data file.

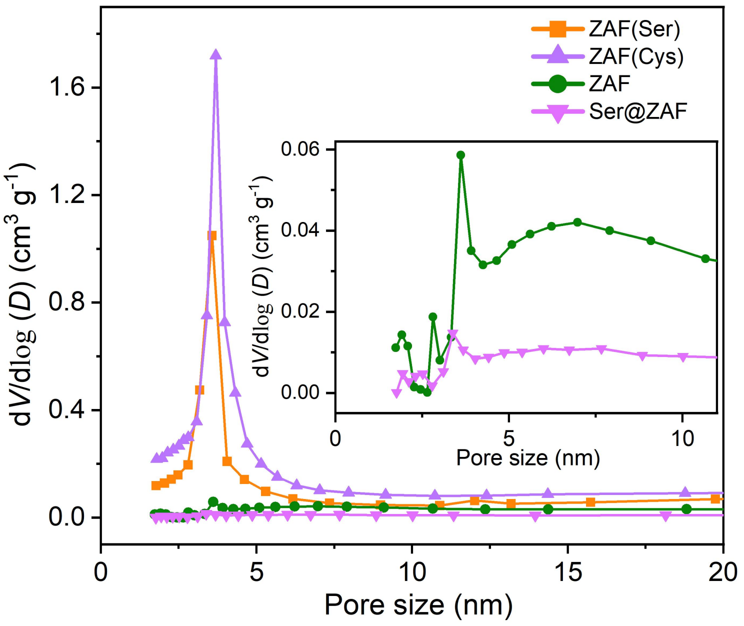

Supplementary Figure 19. Pore size distribution of ZAF, ZAF(Ser), ZAF(Cys) and Ser@ZAF, respectively. ZAF(Ser): serine; ZAF(Cys): cysteine. Source data are provided as a Source Data file.

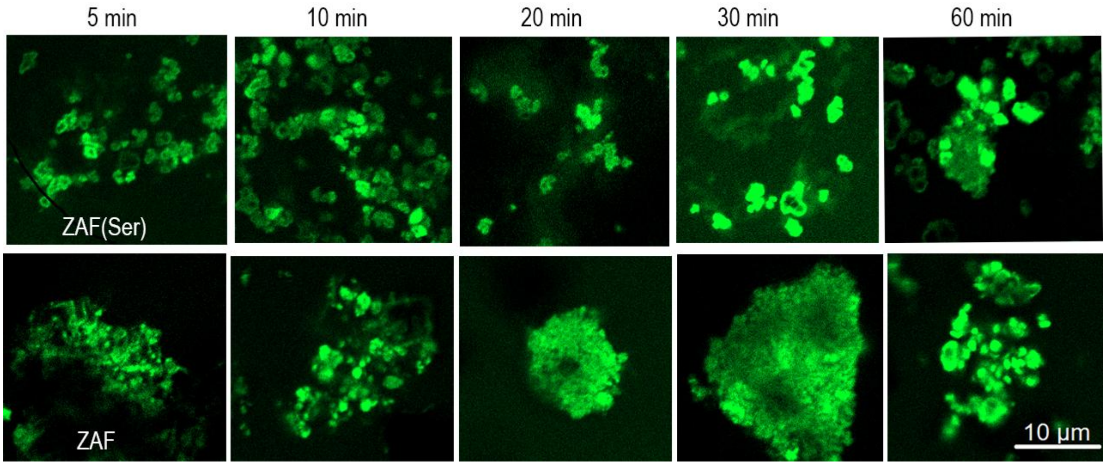

Supplementary Fig. 20. Confocal laser scanning microscope (CLSM) images showing fluorescein isothiocyanate (FITC) entering ZAF and ZAF(Ser) confocal laser after co-incubation for a period of time (5 min, 10 min, 20 min, 30min and 60 min, respectively).

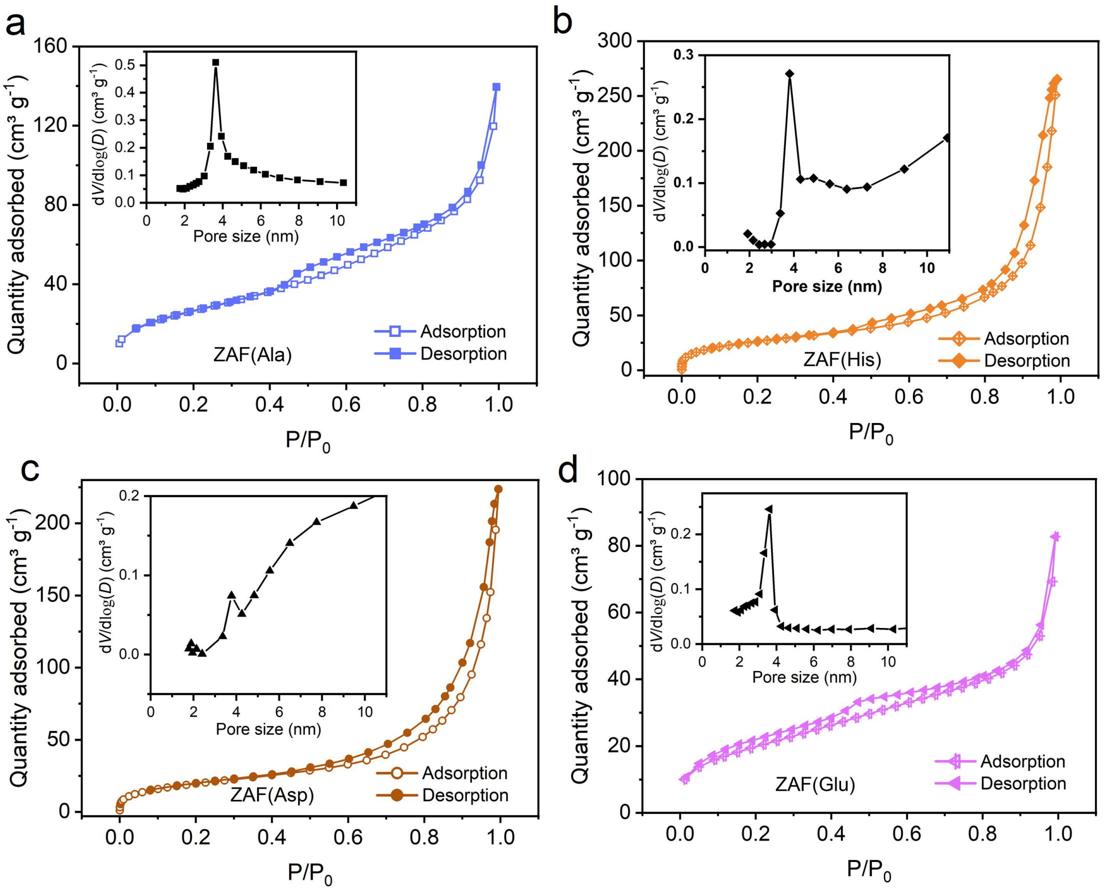

Supplementary Fig. 21. Nitrogen sorption curves and pore size distribution of (a) ZAF(Ala), (b) ZAF(His), (c) ZAF(Asp) and (d) ZAF(Glu). Source data are provided as a Source Data file.

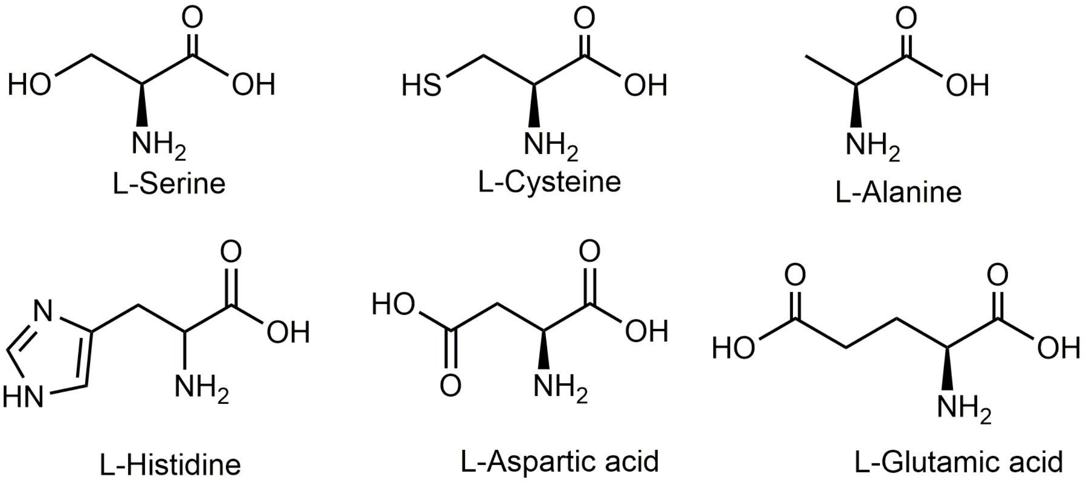

Supplementary Fig. 22. Chemical structure of different amino acids. L-Serine (Ser); L-Cysteine (Cys); L-Alanine (Ala); L-Histidine (His); L-Aspartic acid (Asp); L-Glutamic acid (Glu).

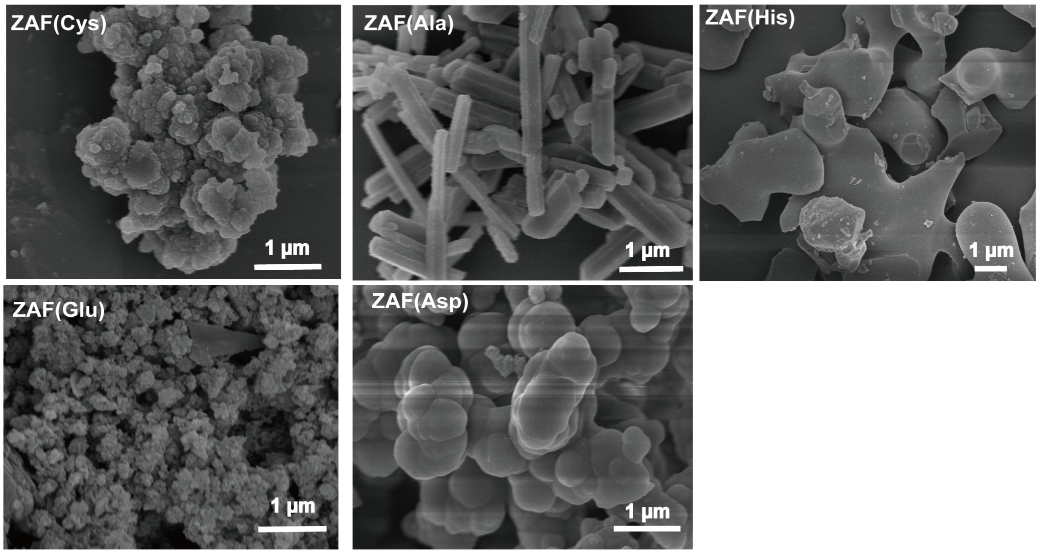

Supplementary Fig. 23. SEM images of ZAF(X) synthesized following the same procedure with ZAF(Ser) except by replacing the serine solution with other amino acid solution. ZAF(Cys): cysteine; ZAF(Ala): alanine; ZAF(His): histidine; ZAF(Asp): aspartic acid; ZAF(Glu): glutamic acid.

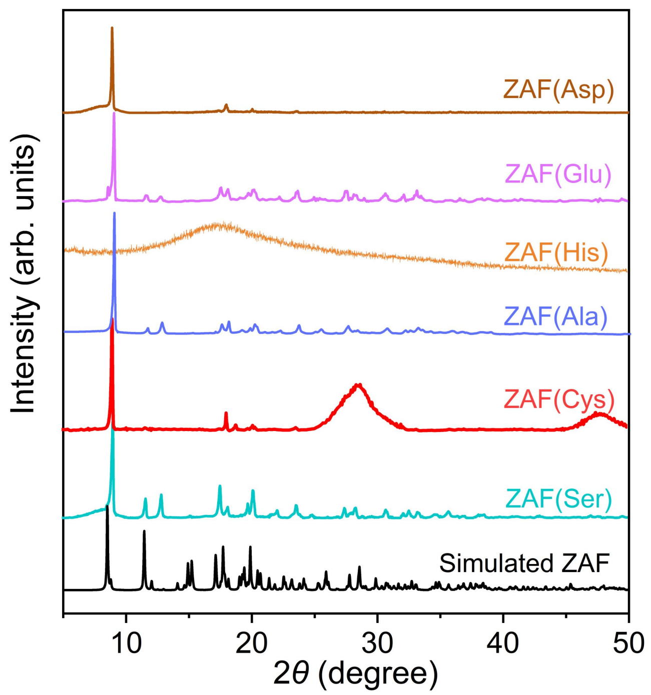

Supplementary Fig. 24. PXRD patterns of ZAF(x) with varying amino acids as substitute of serine. Source data are provided as a Source Data file. (ZAF(Ala) with synthesis time of 6 days)

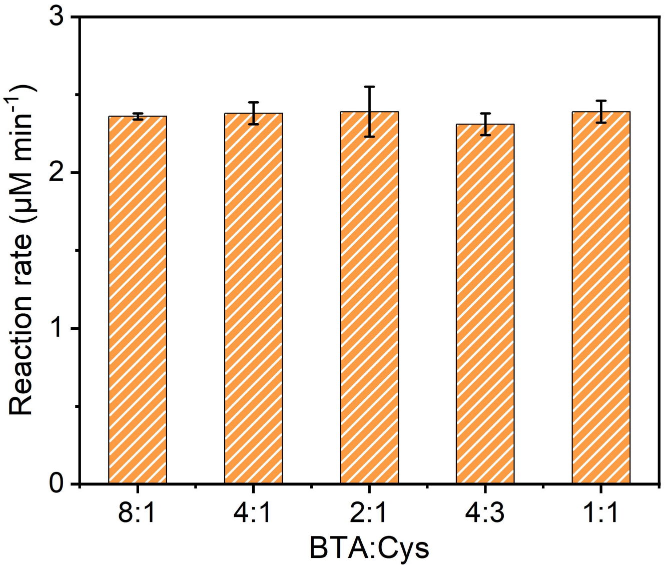

Supplementary Fig. 25. Catalytic activity of ZAF(Cys) obtained with varying the molar ratios of benzotriazole (BTA) to cysteine (Cys) during the synthetic process. Data were represented as mean ± SD (n = 3). Source data are provided as a Source Data file.

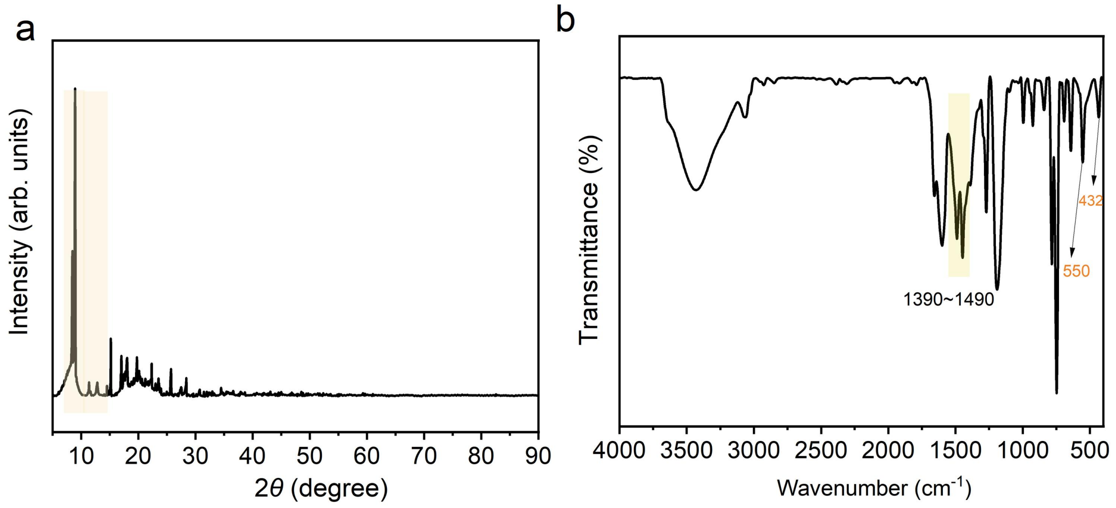

Supplementary Fig. 26. PXRD pattern (a) and FT-IR spectrum of ZAF(Ser) obtained with mixture of DMF and D_2O instead of H_2O as solvents during the synthetic process. Source data are provided as a Source Data file.

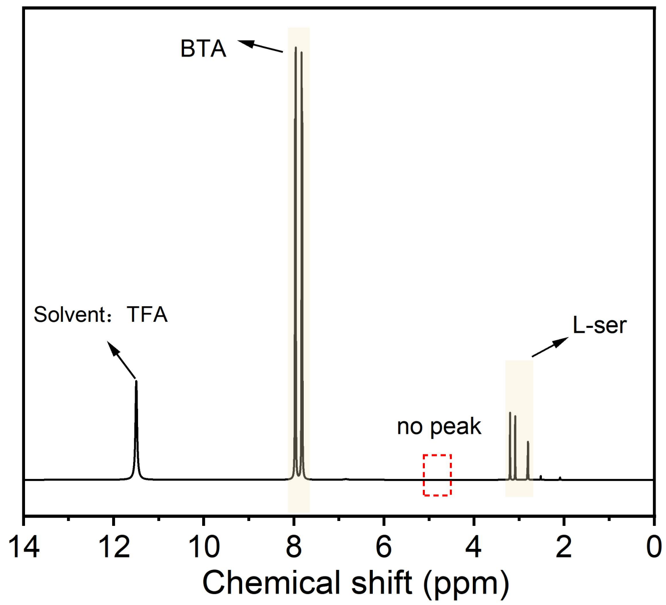

Supplementary Fig. 27. The ^{1}H NMR spectra of ZAF(Ser) obtained with mixture of DMF and deuterium oxide (D_2O) instead of H_2O as solvents during the synthetic process. Source data are provided as a Source Data file.

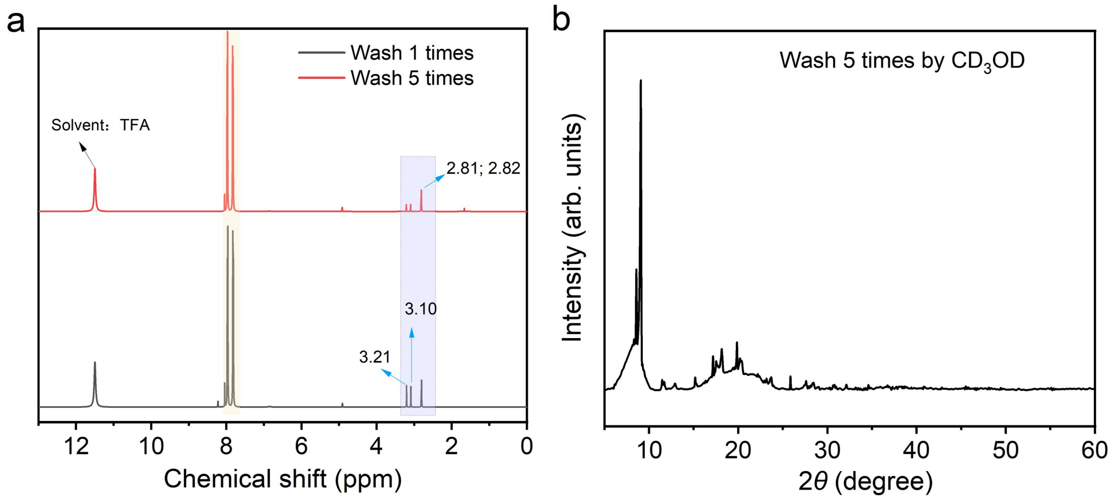

Supplementary Fig. 28. (a) The ^{1}H NMR spectra of ZAF(Ser) obtained by washing with deuterated methanol (CD_3OD) for 1 and 5 times. (b) PXRD pattern of ZAF(Ser) obtained by washing with CD_3OD for 5 times. Source data are provided as a Source Data file.

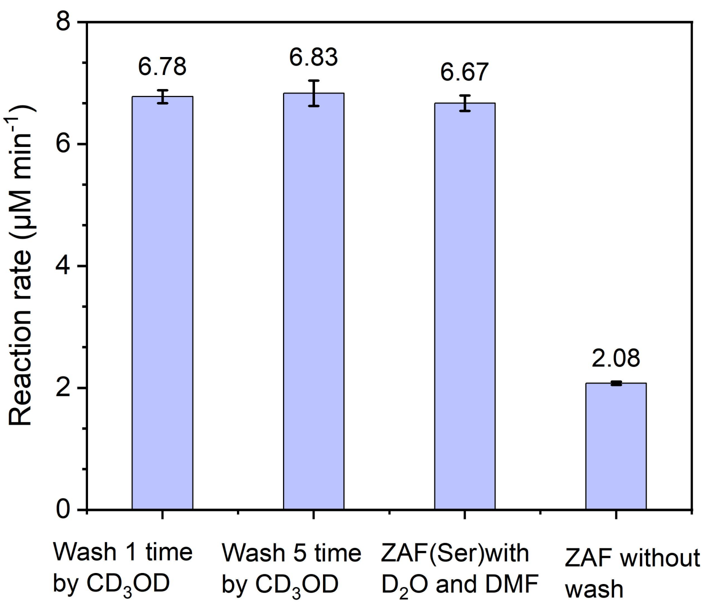

Supplementary Fig. 29. Catalytic activity of ZAF(Ser) via using deuterated methanol (CD_3OD) as the washing solvent, ZAF(Ser) obtained by using deuterium oxide (D_2O) and DMF as the solvent during the synthetic process, ZAF separated from the synthetic system without washing process. Data were represented as mean ± SD (n = 3). Source data are provided as a Source Data file.

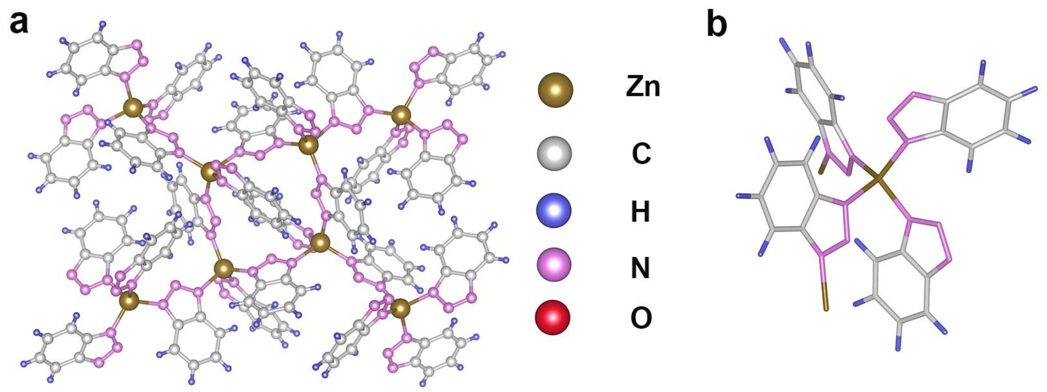

Supplementary Fig. 30. (a) Ball and stick model structure of ZAF(Ser) in single crystal analysis. (b) Coordination environment of divalent zinc atoms. The red, light grey, blue, deep yellow and carmine, orange spheres represent O, C, H, Zn and N atoms, respectively.

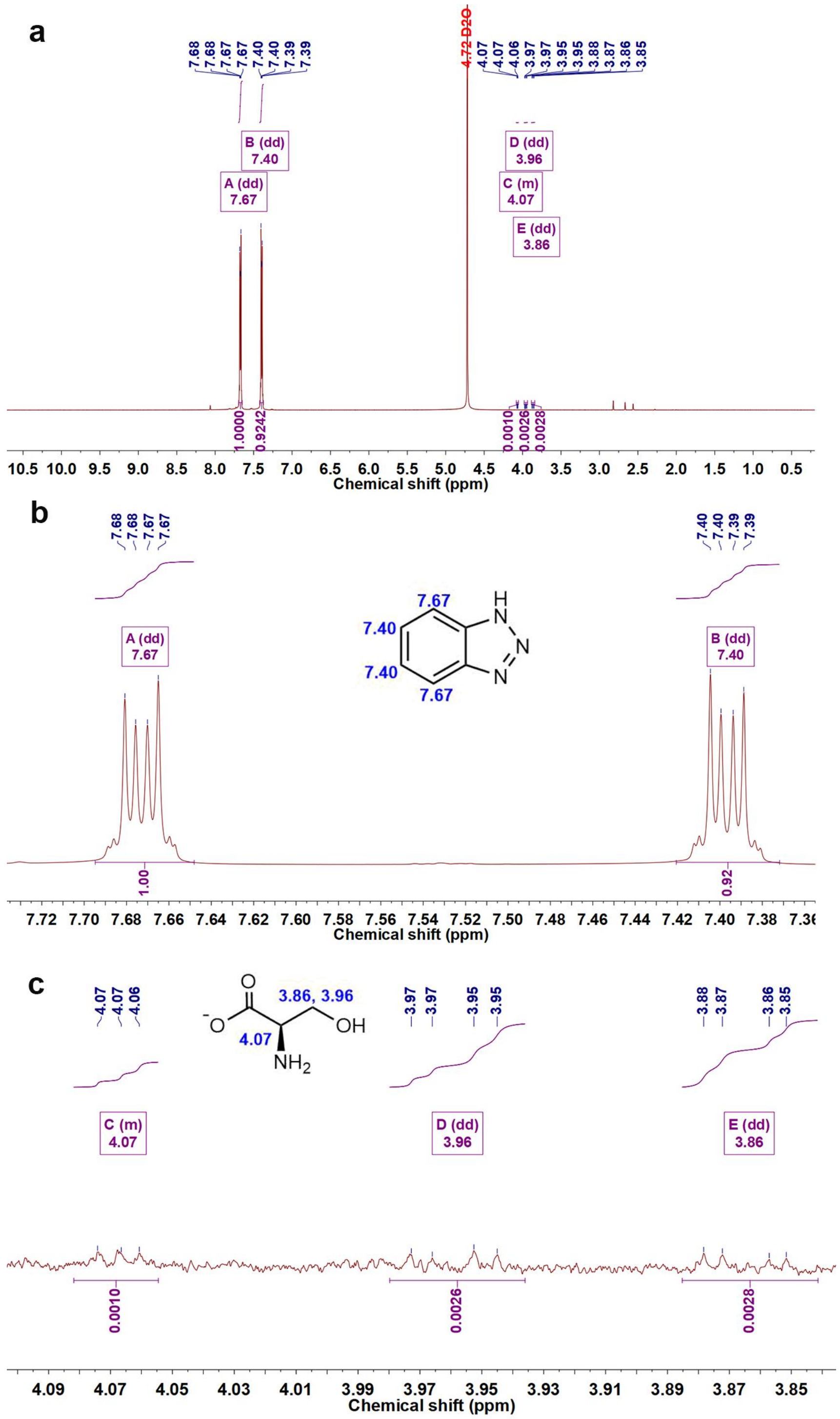

Supplementary Figure 31. ^{1}H NMR of ZAF(Ser) single crystal. (b) Partially enlarged ^{1}H NMR spectrum of benzotriazole (BTA). (c) Partially enlarged ^{1}H NMR spectrum of serine (Ser).

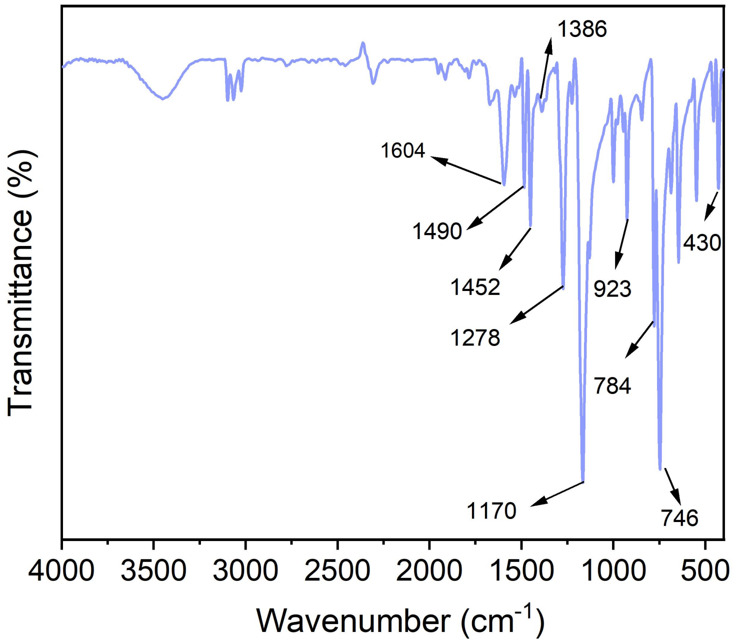

Supplementary Fig. 32. FT-IR spectra of ZAF(Ser) crystal. Source data are provided as a Source Data file.

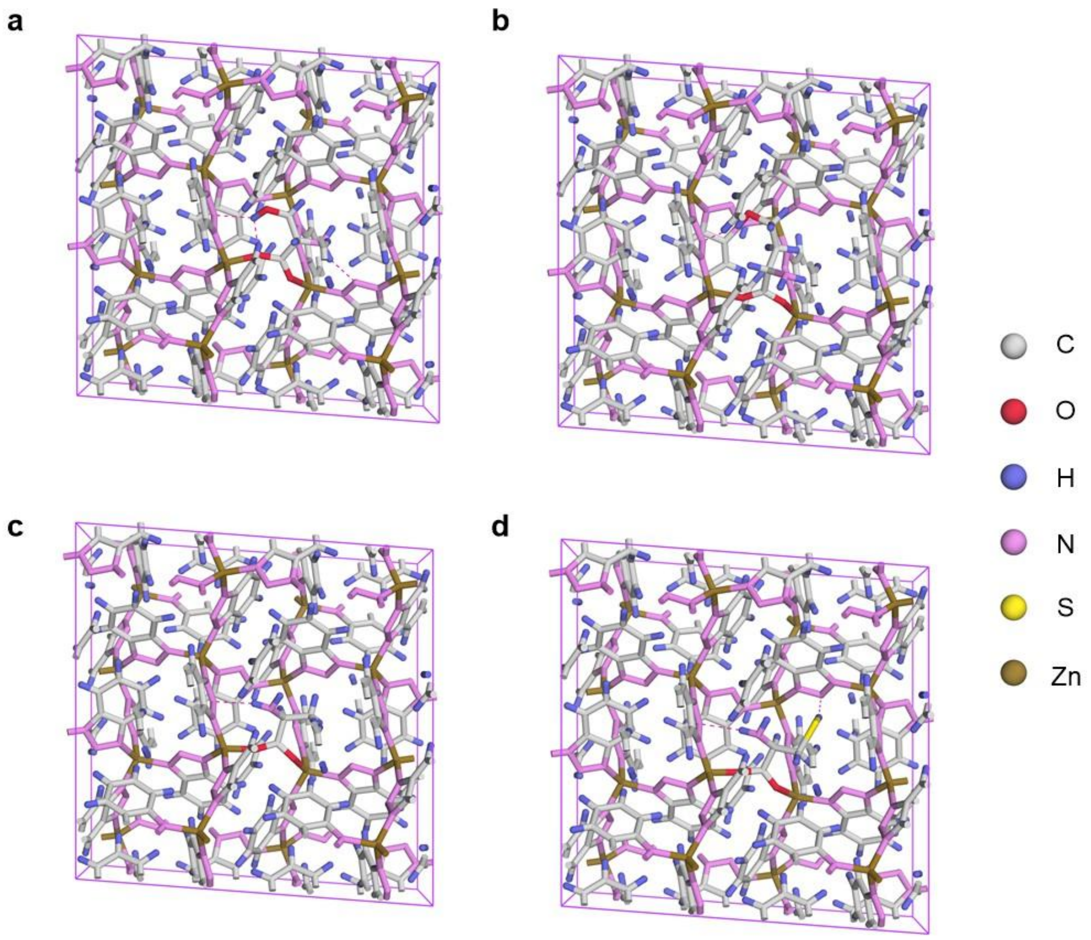

Supplementary Figure 33. The models of (a) serine, (b) homoserine, (c) alanine, and (d) cysteine coordinated MOFs. The red, light grey, blue, deep yellow, carmine and yellow, orange spheres represent O, C, H, Zn, N, and S atoms, respectively. The red dashed line indicates the hydrogen bonding.

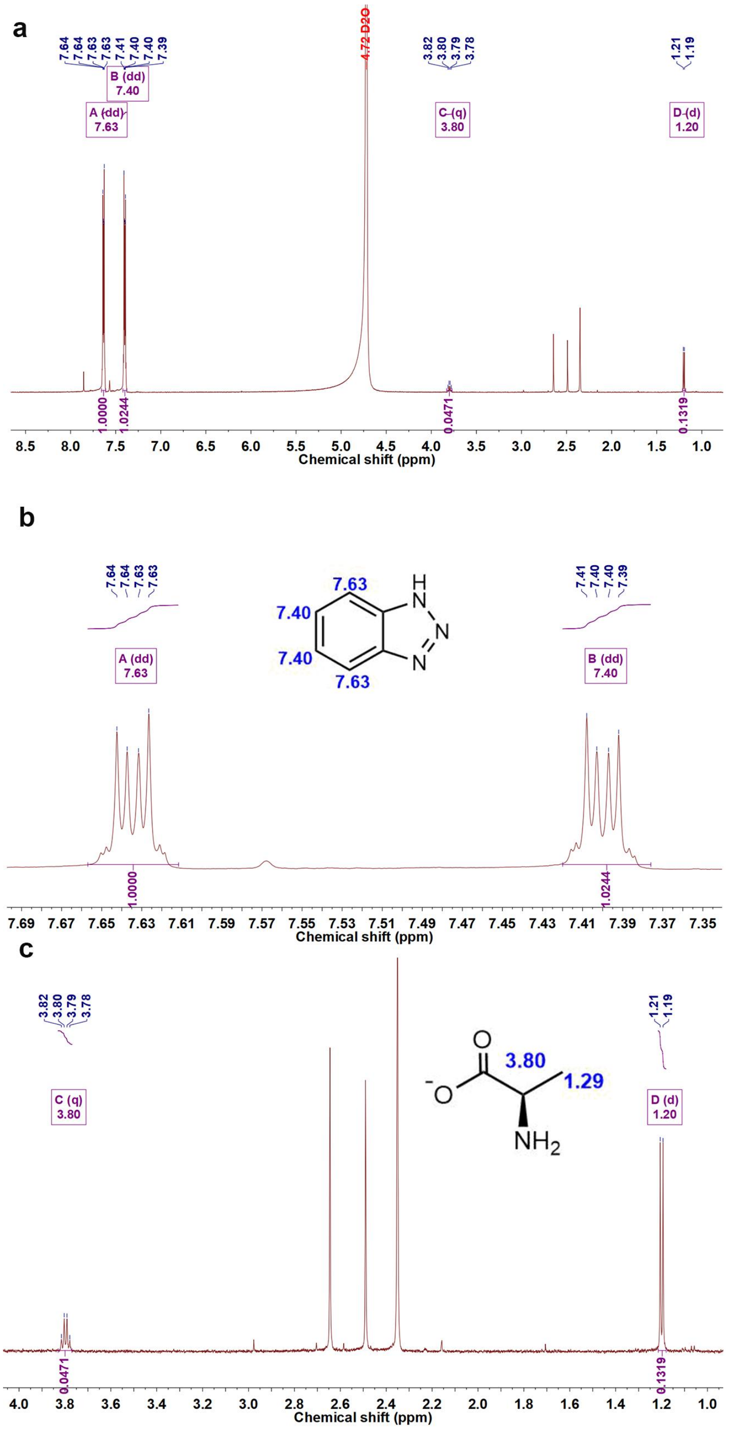

Supplementary Fig. 34. ^{1}H NMR of ZAF(Ala). (b) Partially enlarged ^{1}H NMR spectrum of benzotriazole (BTA). (c) Partially enlarged ^{1}H NMR spectrum of alanine (Ala).

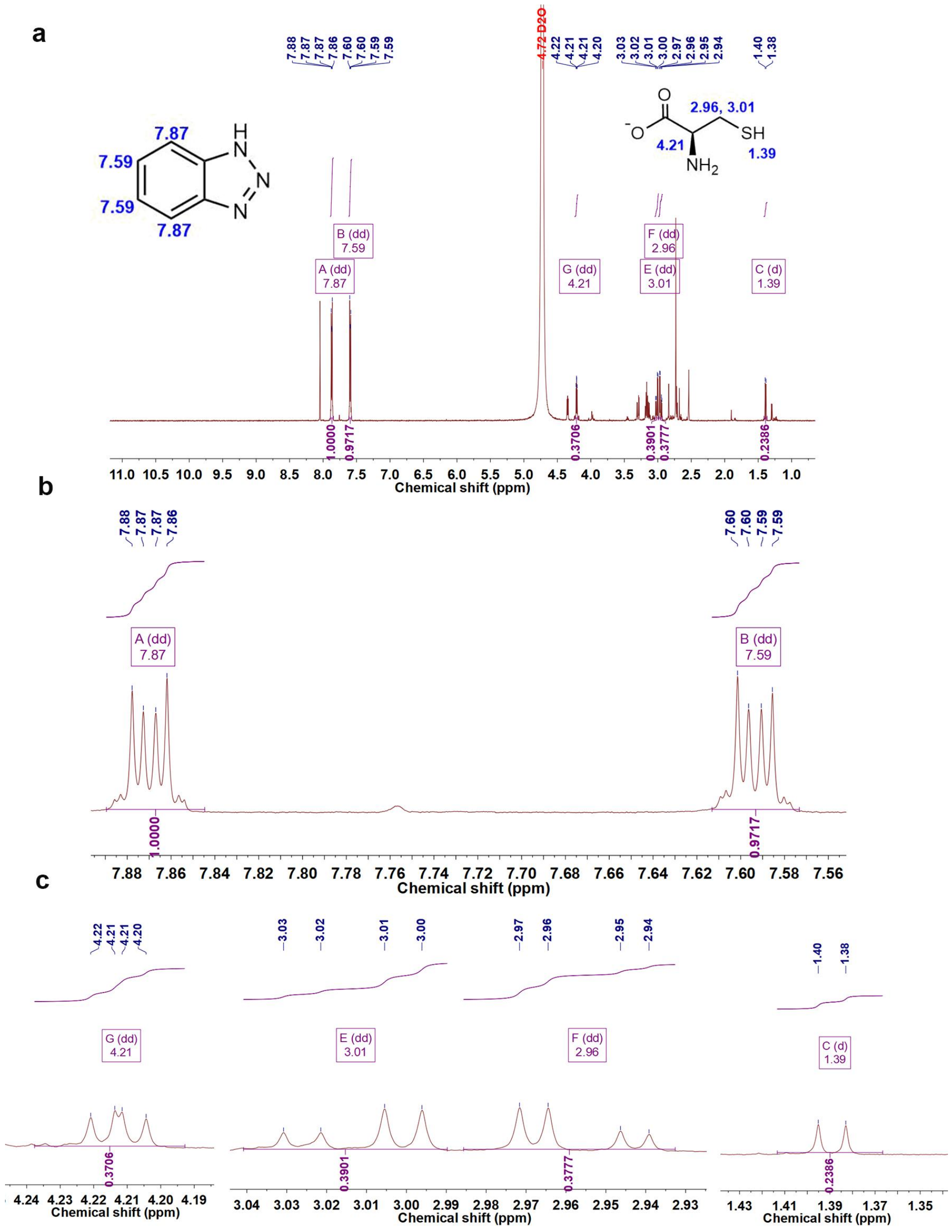

Supplementary Fig. 35. ^{1}H NMR of ZAF(Cys). (b) Partially enlarged ^{1}H NMR spectrum of benzotriazole (BTA). (c) Partially enlarged ^{1}H NMR spectrum of cysteine (Cys).

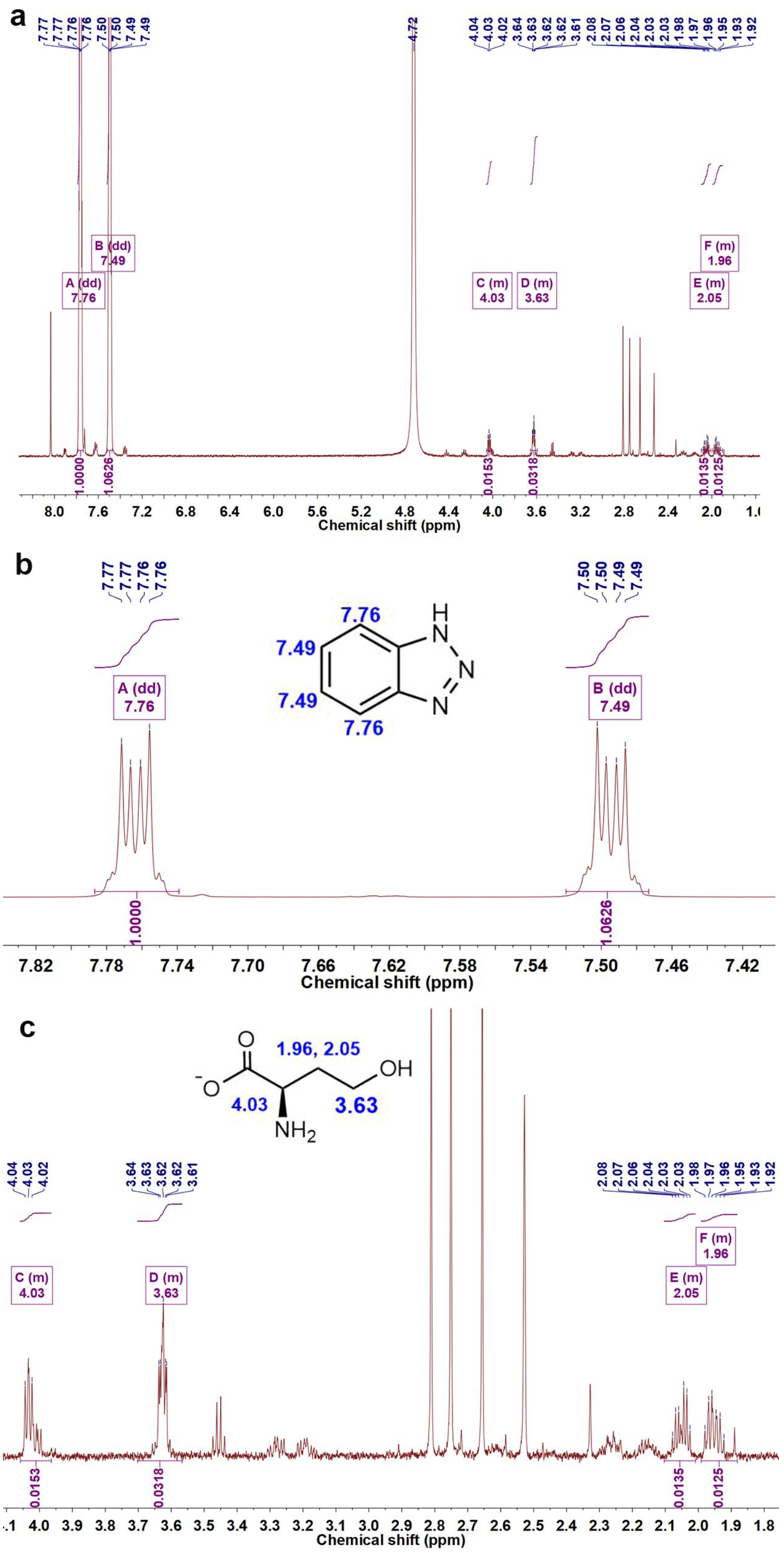

Supplementary Fig. 36. ^{1}H NMR of ZAF(HSer). (b) Partially enlarged ^{1}H NMR spectrum of benzotriazole (BTA). (c) Partially enlarged ^{1}H NMR spectrum of homoserine (Hser).

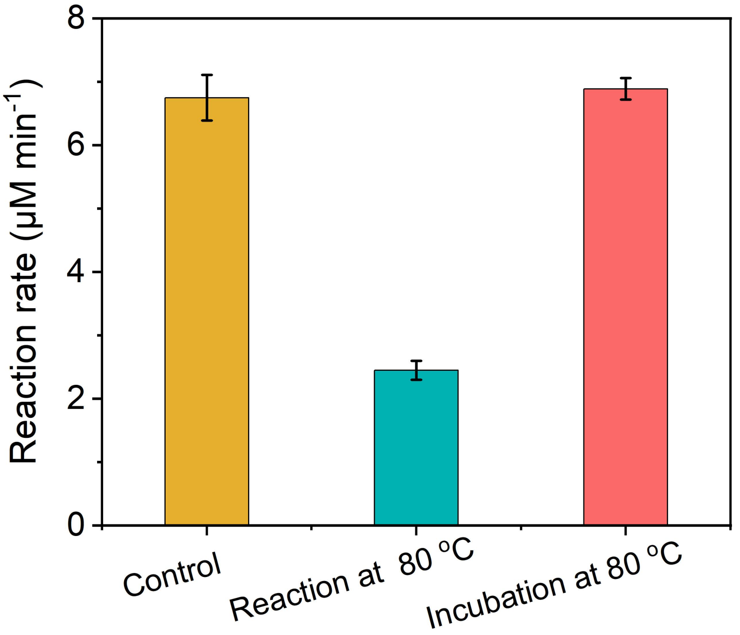

Supplementary Fig. 37. Catalytic activity of ZAF(Ser) at room temperature (100% referece), ZAF(Ser) with reaction temperature at 80 °C (reaction at 80 °C), and incubation of ZAF(Ser) at 80 °C and then cooled down to room temperature followed by enzymatic assay (incubated at 80 °C). Data were represented as mean ± SD (n = 3). Source data are provided as a Source Data file.

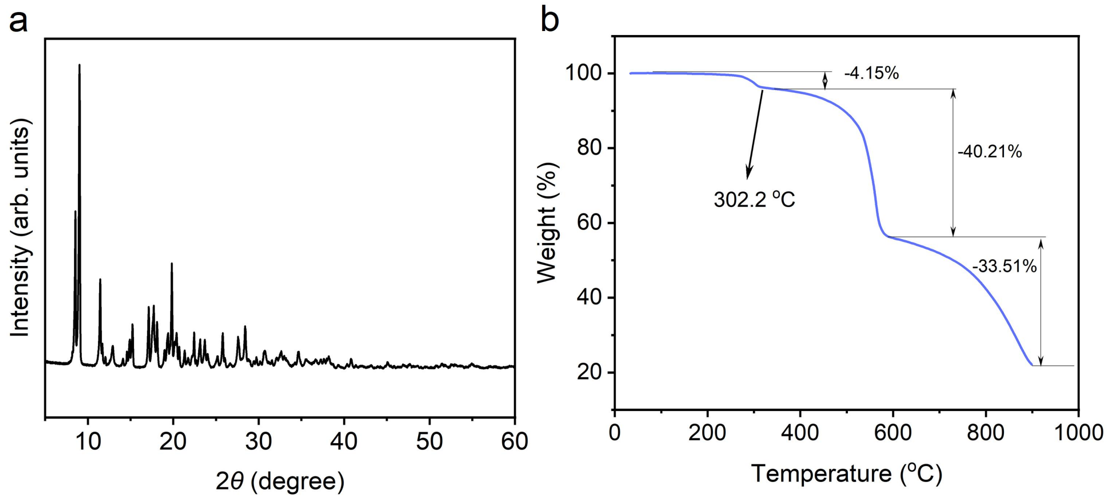

Supplementary Fig. 38. (a) PXRD pattern and (b) TGA analysis of ZAF(Ser) after high-temperature reaction. Source data are provided as a Source Data file.

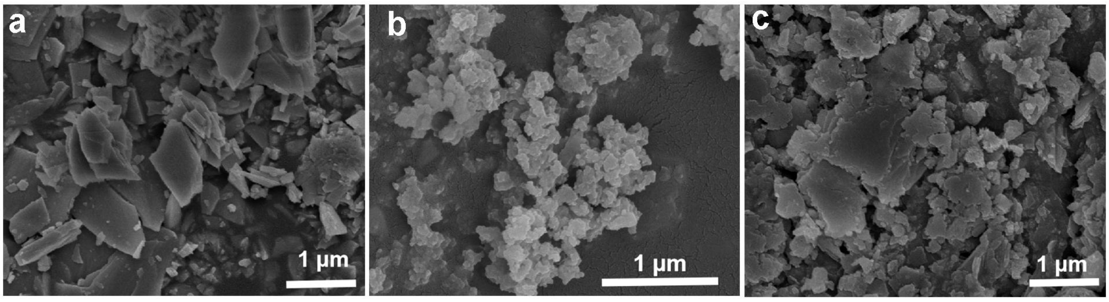

Supplementary Fig. 39. SEM images of (a) ZnSer, (b) ZAF, (c) physical mixture of ZnSer and ZAF nanoparticles (ZAF+ZnSer).

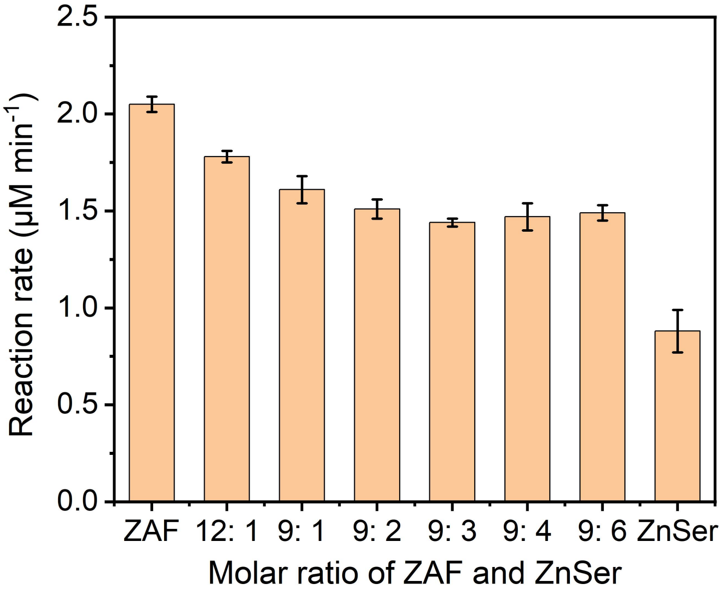

Supplementary Fig. 40. Catalytic activity of physical mixture of ZAF and ZnSer complex with different molar ratio of ZAF to ZnSer. Data were represented as mean ± SD (n = 3). Source data are provided as a Source Data file.

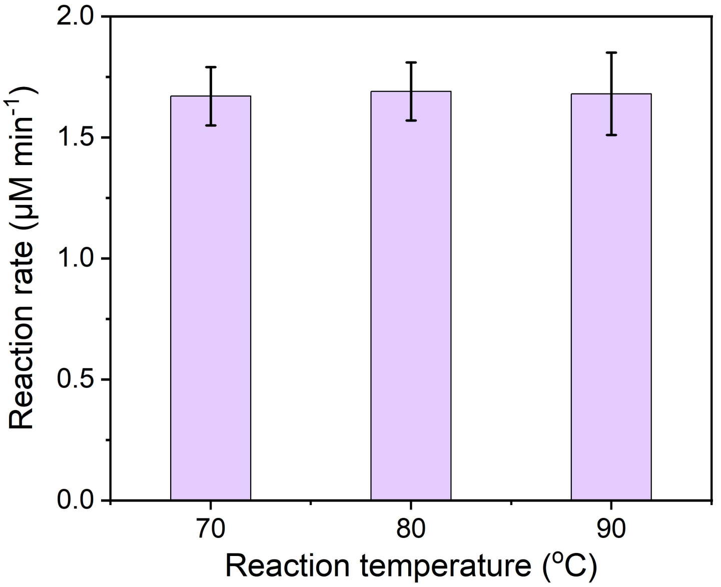

Supplementary Fig. 41. Catalytic activity of physical mixture of ZAF and ZnSer with high reaction temperature. Data were represented as mean ± SD (n = 3). Source data are provided as a Source Data file.

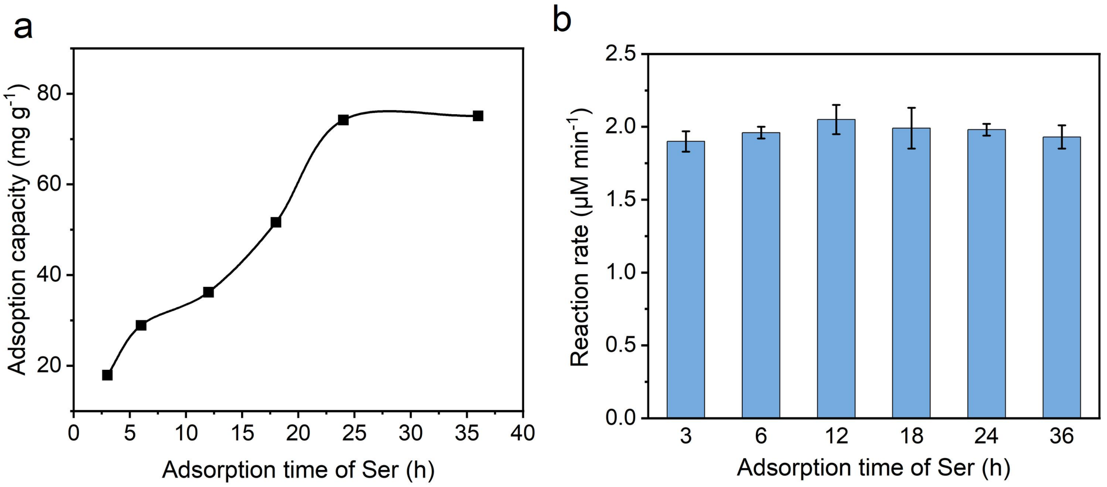

Supplementary Fig. 42. (a) Adsorption capacity of Ser for ZAF with different adsorption time. (b) Reaction rate of ZAF with different amount of Ser adsorbed (denoted as Ser/ZAF). Data were represented as mean ± SD (n = 3). Source data are provided as a Source Data file.

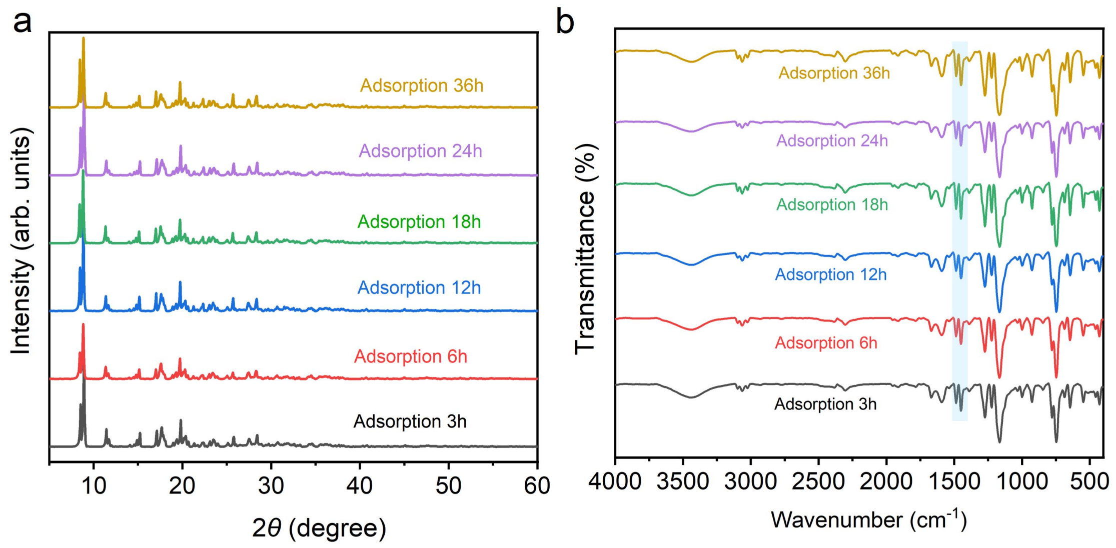

Supplementary Fig. 43. (a) PXRD patterns and (b) FT-IR spectra of Ser/ZAF with different adsorption time. Source data are provided as a Source Data file.

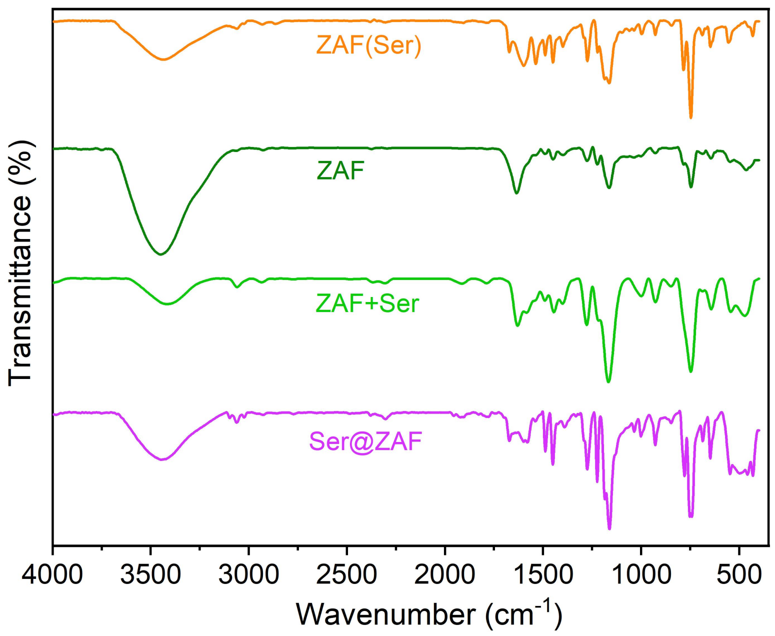

Supplementary Fig. 44. FT-IR spectra of ZAF(Ser), ZAF, physically mixture of ZAF and serine (ZAF+Ser), and Ser@ZAF obtained via post-modification method. Source data are provided as a Source Data file.

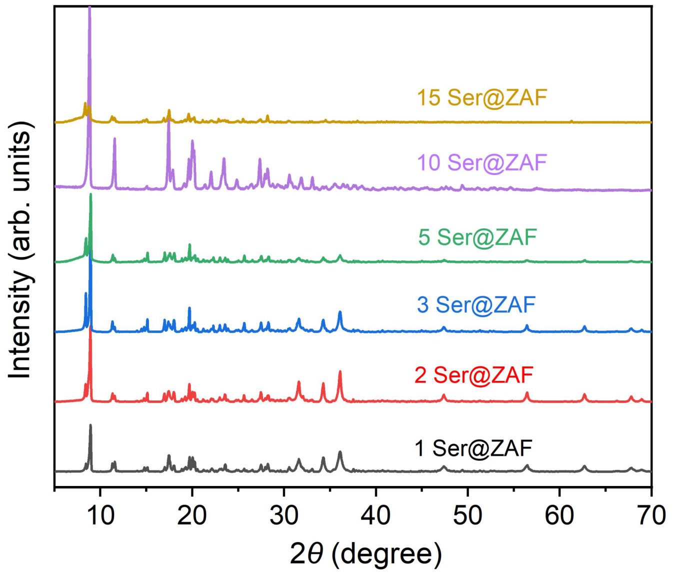

Supplementary Fig. 45. The PXRD patterns of Ser@ZAF by doping different mole amounts of serine into ZAF. (xSer@ZAF: x mmol serine added when 1g ZAF was used during the post-synthetic modification process). Source data are provided as a Source Data file.

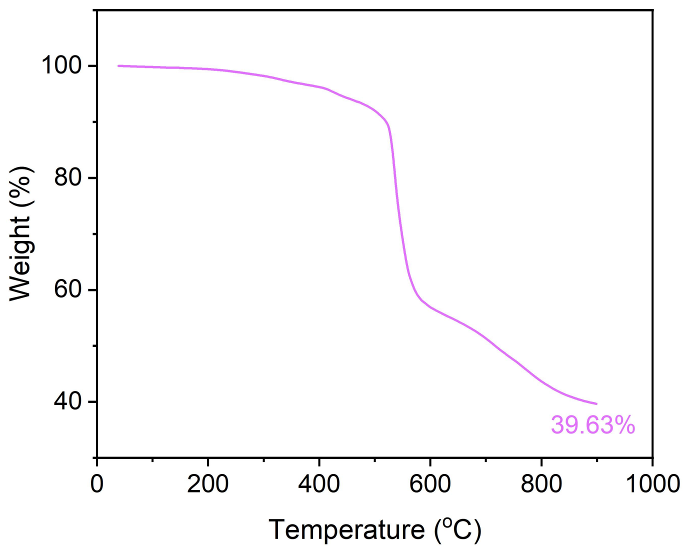

Supplementary Fig. 46. Thermogravimetric analysis (TGA) Curve of Ser@ZAF. Source data are provided as a Source Data file.

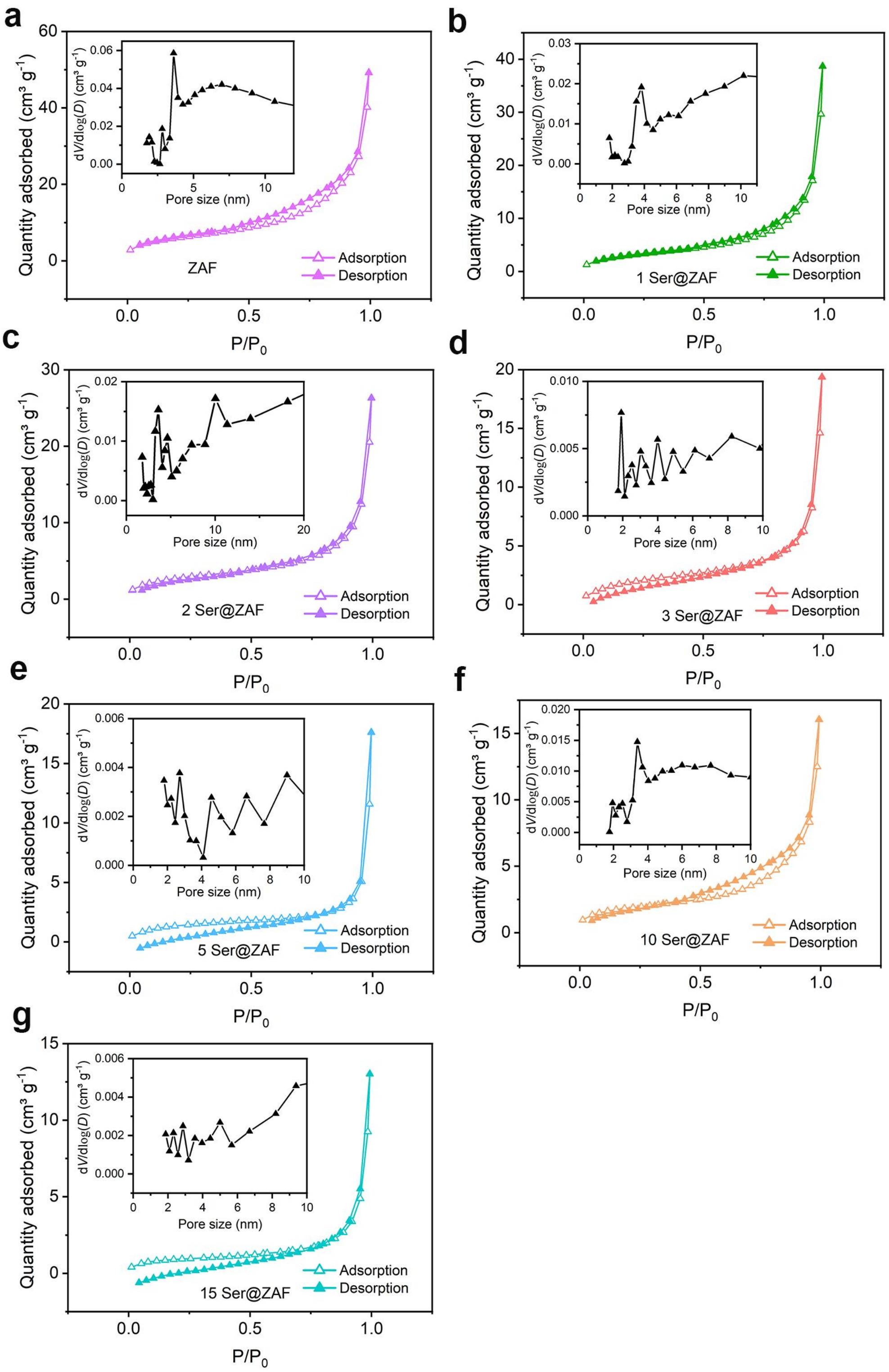

Supplementary Figure 47. Nitrogen sorption isotherms and pore size distribution for xSer@ZAF obtained via post-synthetic modification. Source data are provided as a Source Data file.

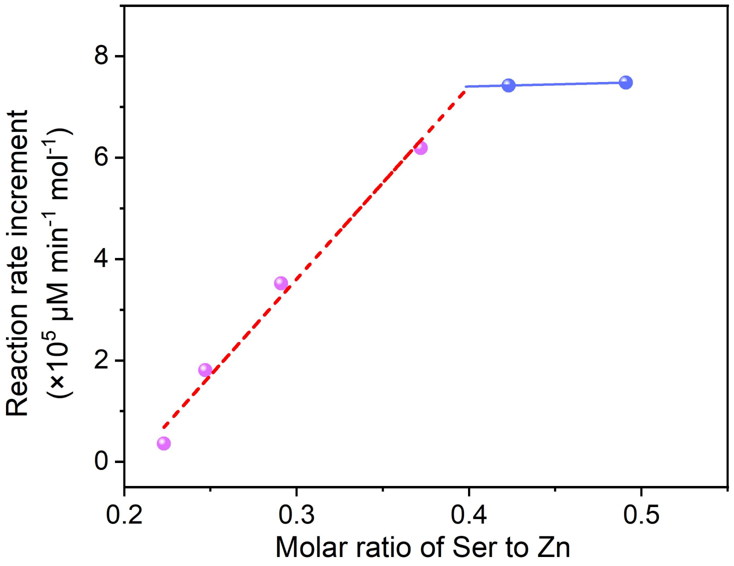

Supplementary Fig. 48. Quantification relationship of the activity contribution of incorporated Ser with molar ratio of Ser to Zn in xSer@ZAF under identical amount of Zn. Source data are provided as a Source Data file.

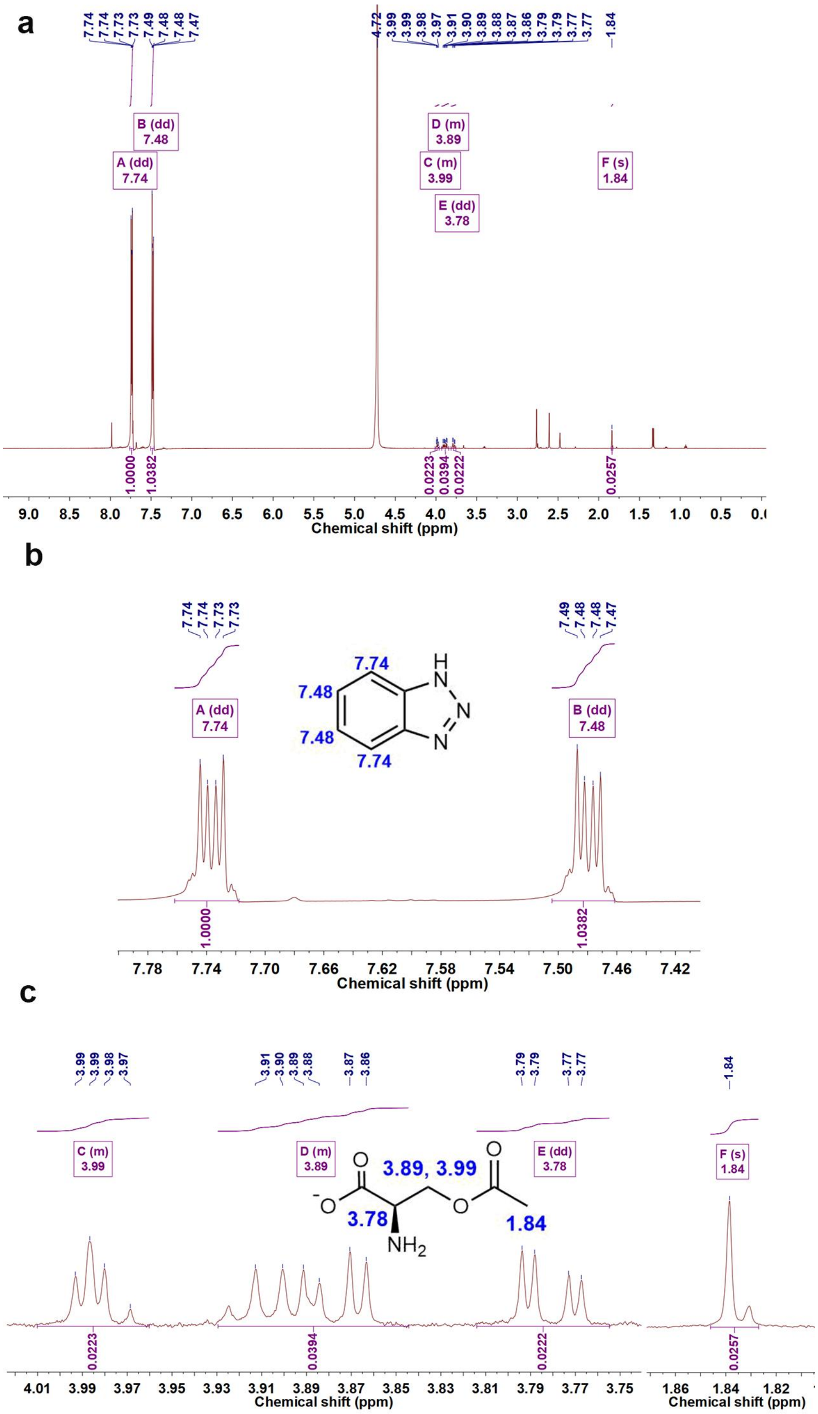

Supplementary Fig. 49. (a) ^{1}H NMR of acid digested ZAF(O-Ac-Ser). (b) Partially enlarged ^{1}H NMR spectrum of benzotriazole (BTA). (c) Partially enlarged ^{1}H NMR spectrum of O-acetyl-L-serine (O-Ac-Ser).

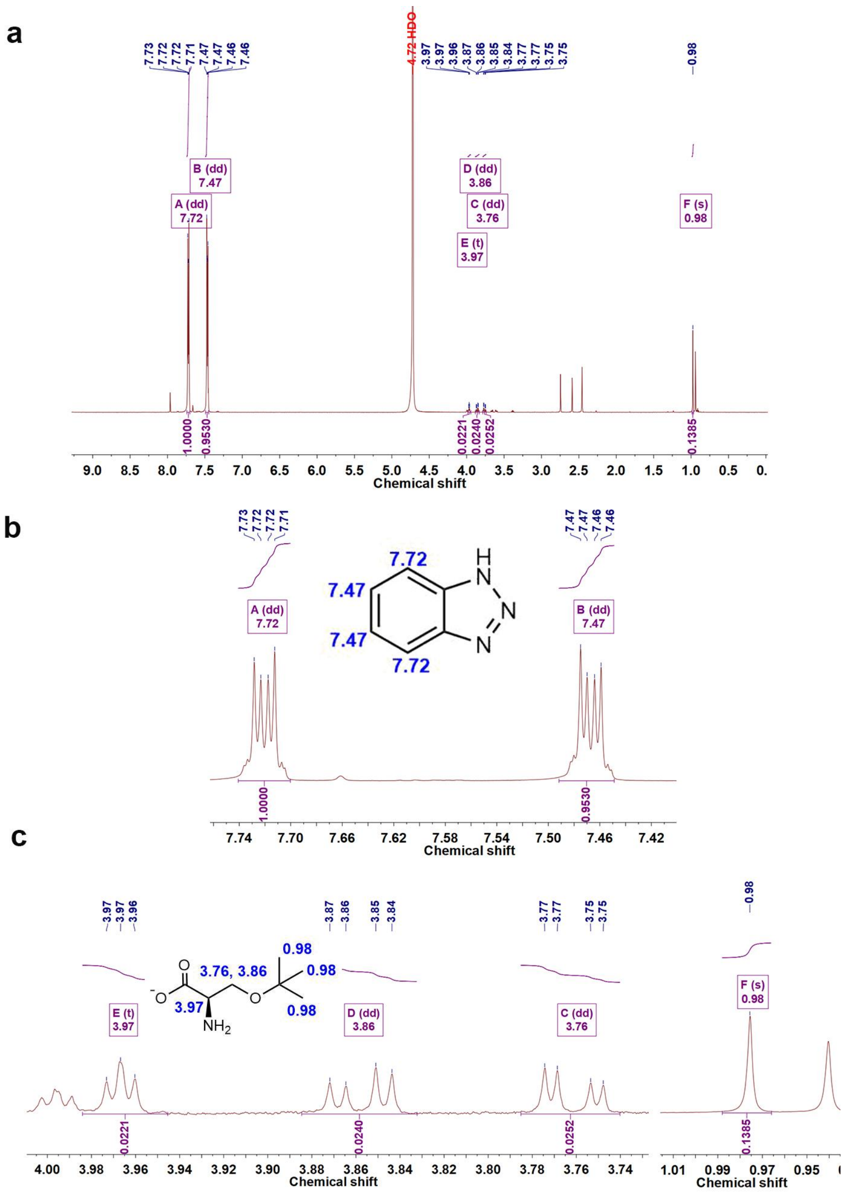

Supplementary Fig. 50. (a) ^{1}H NMR of acid digested ZAF(O-tBu-Ser). (b) Partially enlarged ^{1}H NMR spectrum of benzotriazole (BTA). (c) Partially enlarged ^{1}H NMR spectrum of O-tert-butyl-L-serine (O-tBu-Ser).

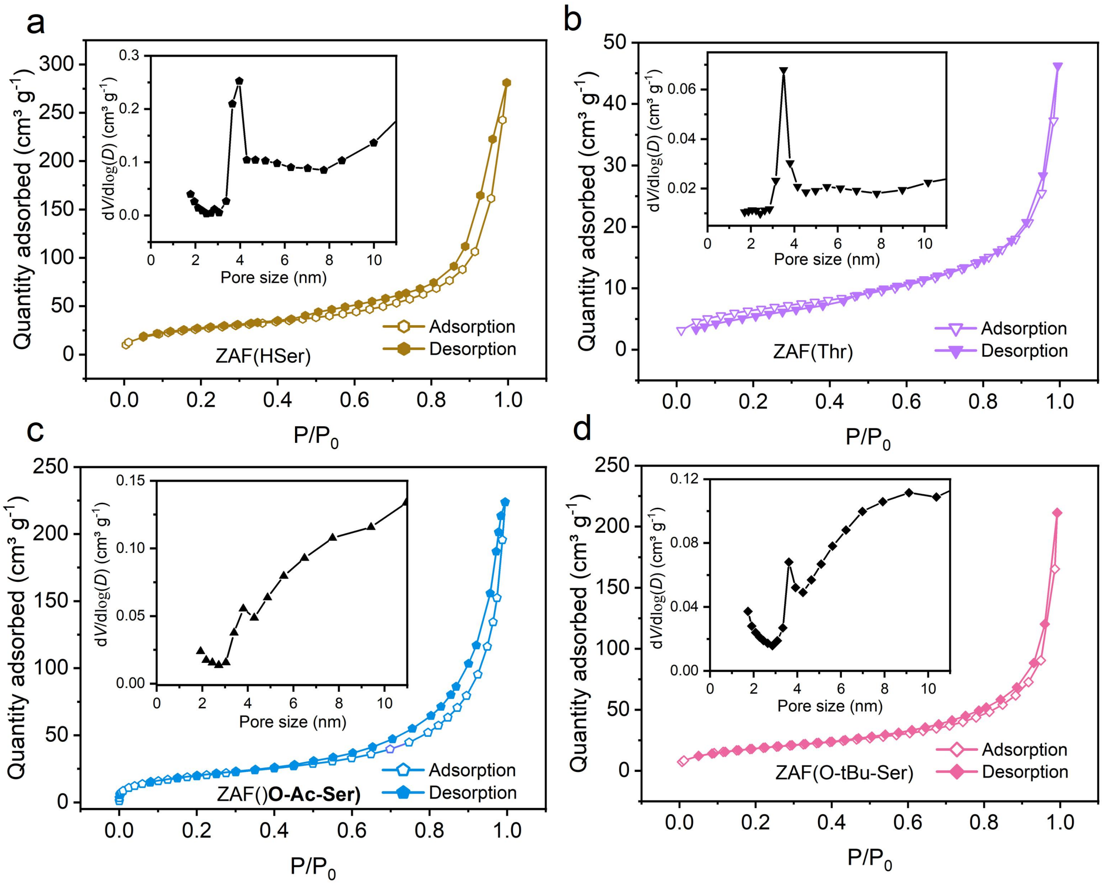

Supplementary Fig. 51. Nitrogen sorption curves and pore size distribution of (a) ZAF(HSer), (b) ZAF(Thr), (c) ZAF(O-Ac-Ser) and (d) ZAF(O-tBu-Ser). Hser: L-homoserine; Thr: L-threonine; O-Ac-Ser: O-acetyl-L-serine; O-tBu-Ser: O-tert-butyl-L-serine. Source data are provided as a Source Data file.

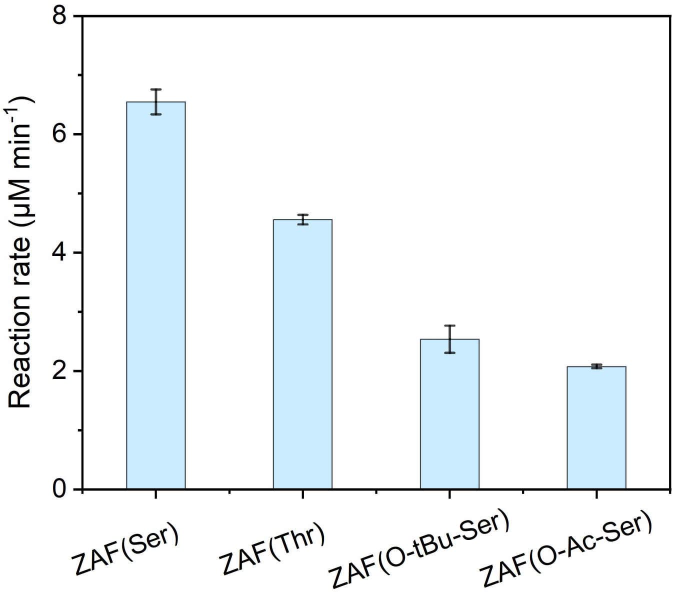

Supplementary Fig. 52. Catalytic activity of ZAF(Ser), ZAF(Thr), ZAF(O-tBu-Ser), and ZAF(O-Ac-Ser). Hser: L-homoserine; Thr: L-threonine; O-Ac-Ser: O-acetyl-L-serine; O-tBu-Ser: O-tert-butyl-L-serine. Data were represented as mean ± SD (n = 3). Source data are provided as a Source Data file.

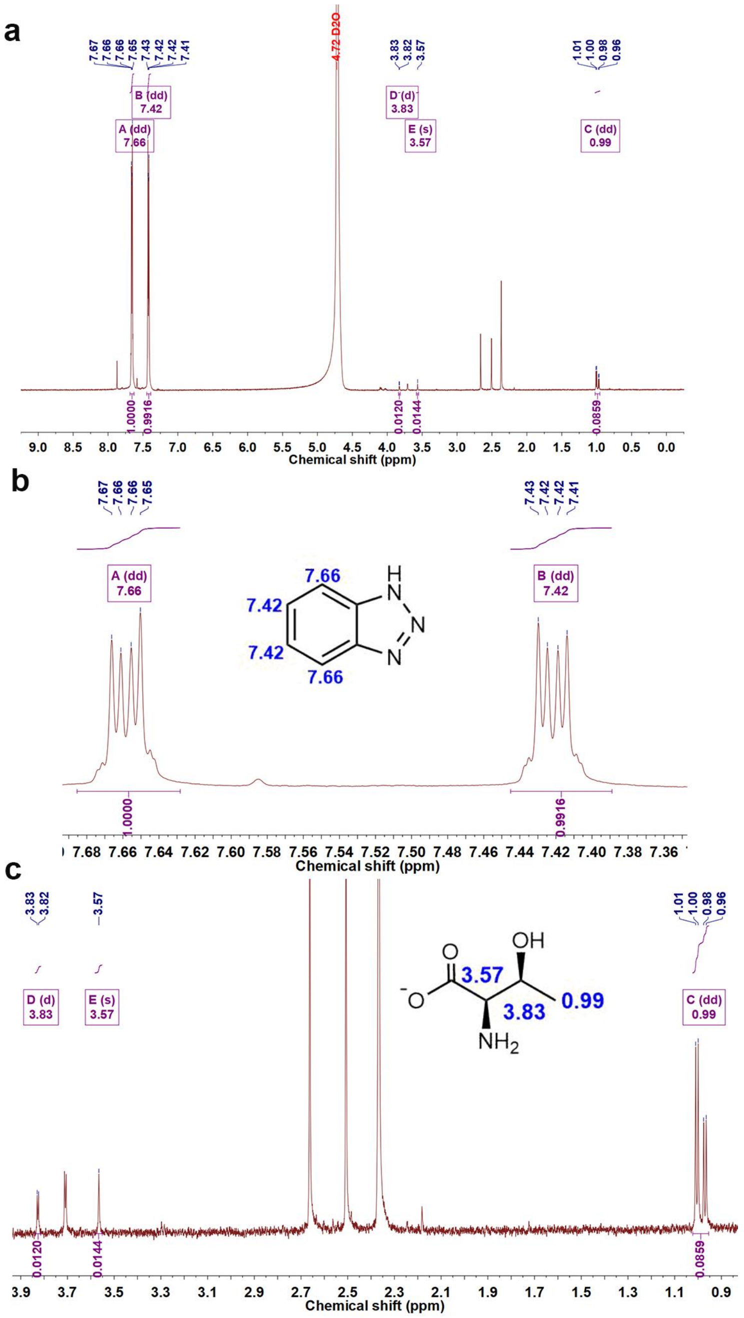

Supplementary Fig. 53. (a) ^{1}H NMR of acidolysis ZAF(Thr). (b) Partially enlarged ^{1}H NMR spectrum of benzotriazole (BTA). (c) Partially enlarged ^{1}H NMR spectrum of threonine (Thr).

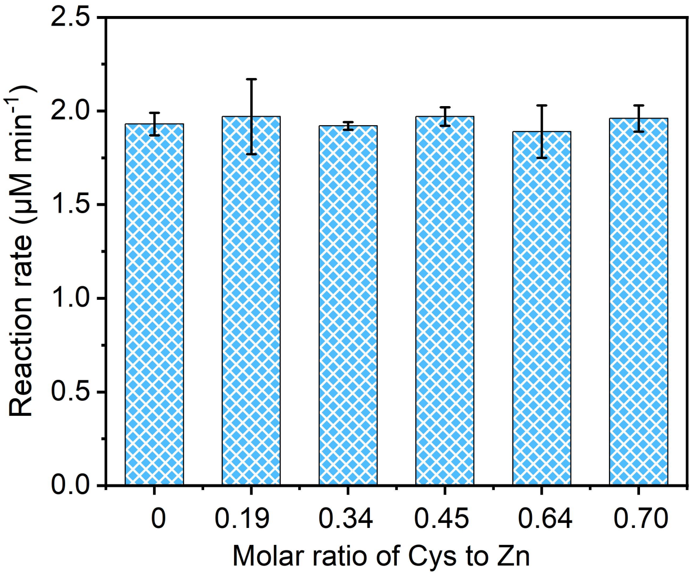

Supplementary Fig. 54. Catalytic activity of xCys@ZAF obtained via post-synthetic modification method. Data were represented as mean ± SD (n = 3). Source data are provided as a Source Data file.

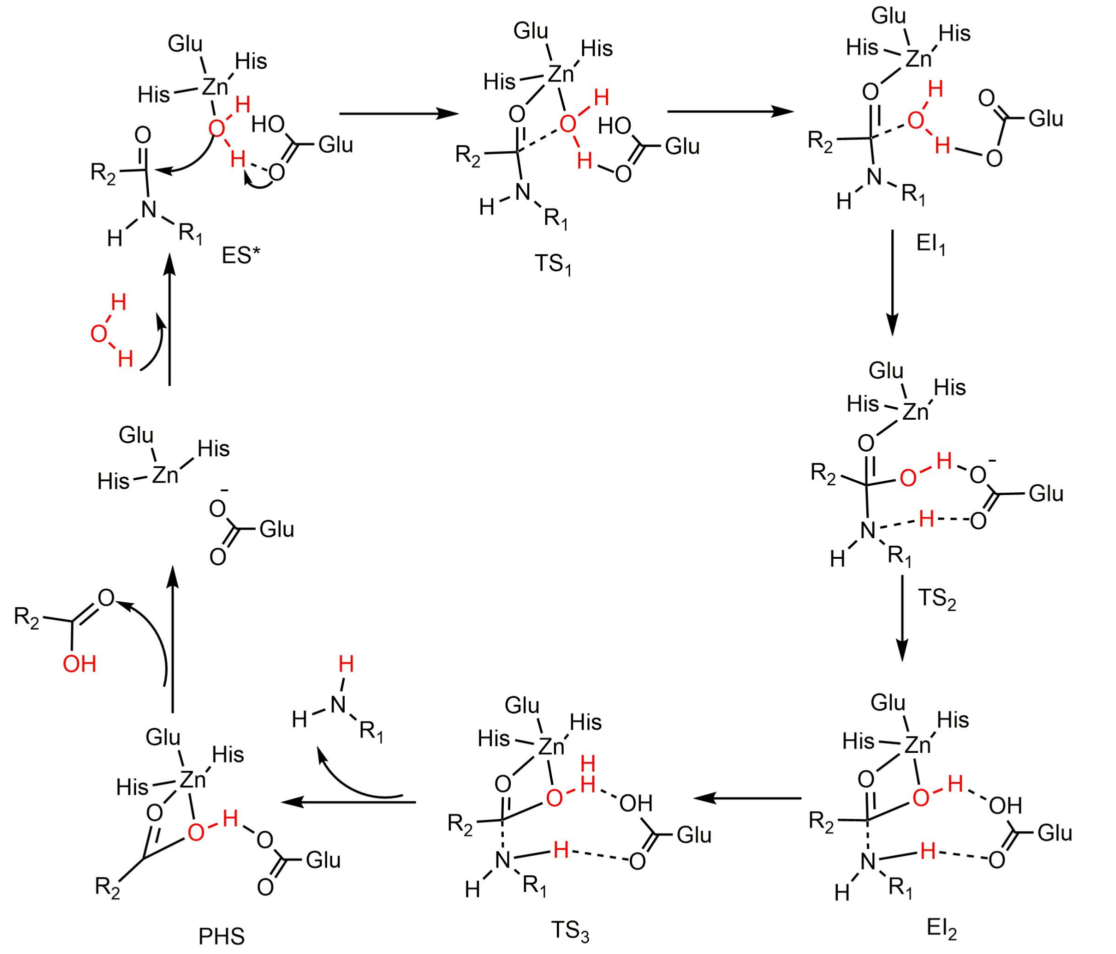

Supplementary Fig. 55. Chemical structures of species along the reaction pathway via Lewis acid mediated process in native metallohydrolase[1, 2].

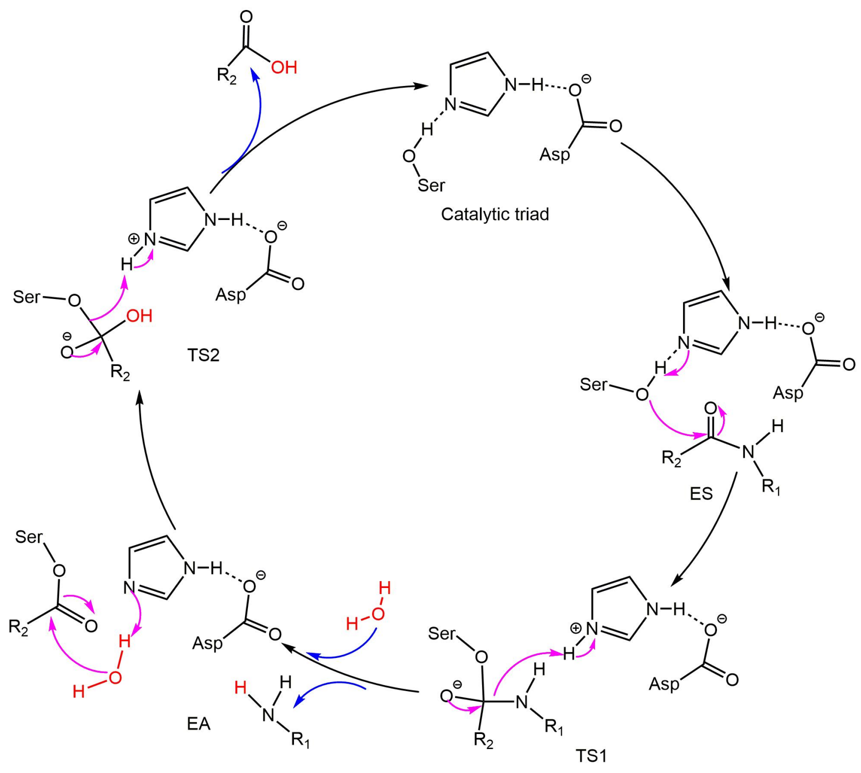

Supplementary Fig. 56. Detailed calculation pathway via hydrogen bonding mediated process based on the Asp-His-Ser triad active site in native hydrolase[3].

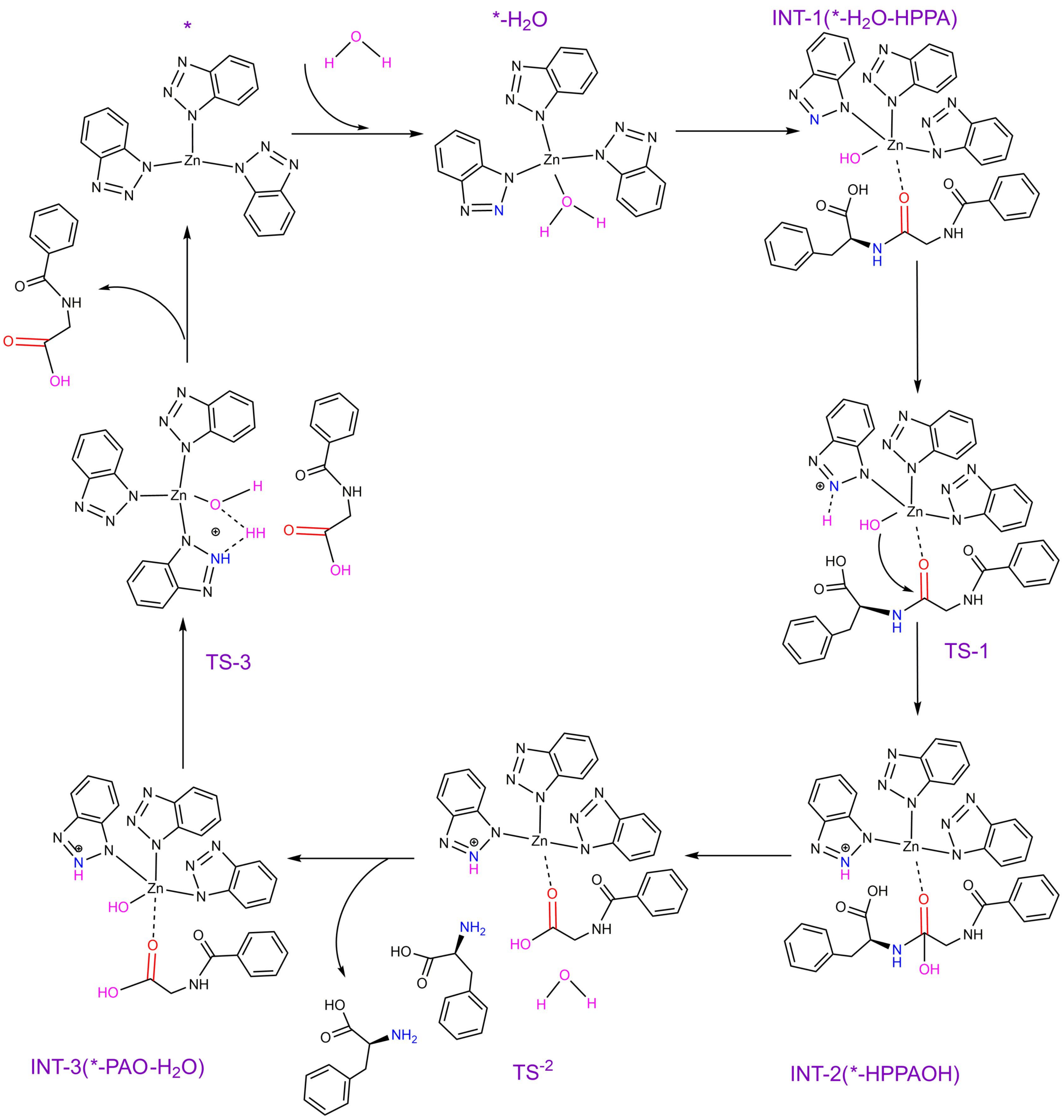

Supplementary Fig. 57. Proposed reaction pathway of ZAF with Lewis acid mediated active site (Zn-OH active site without serine).

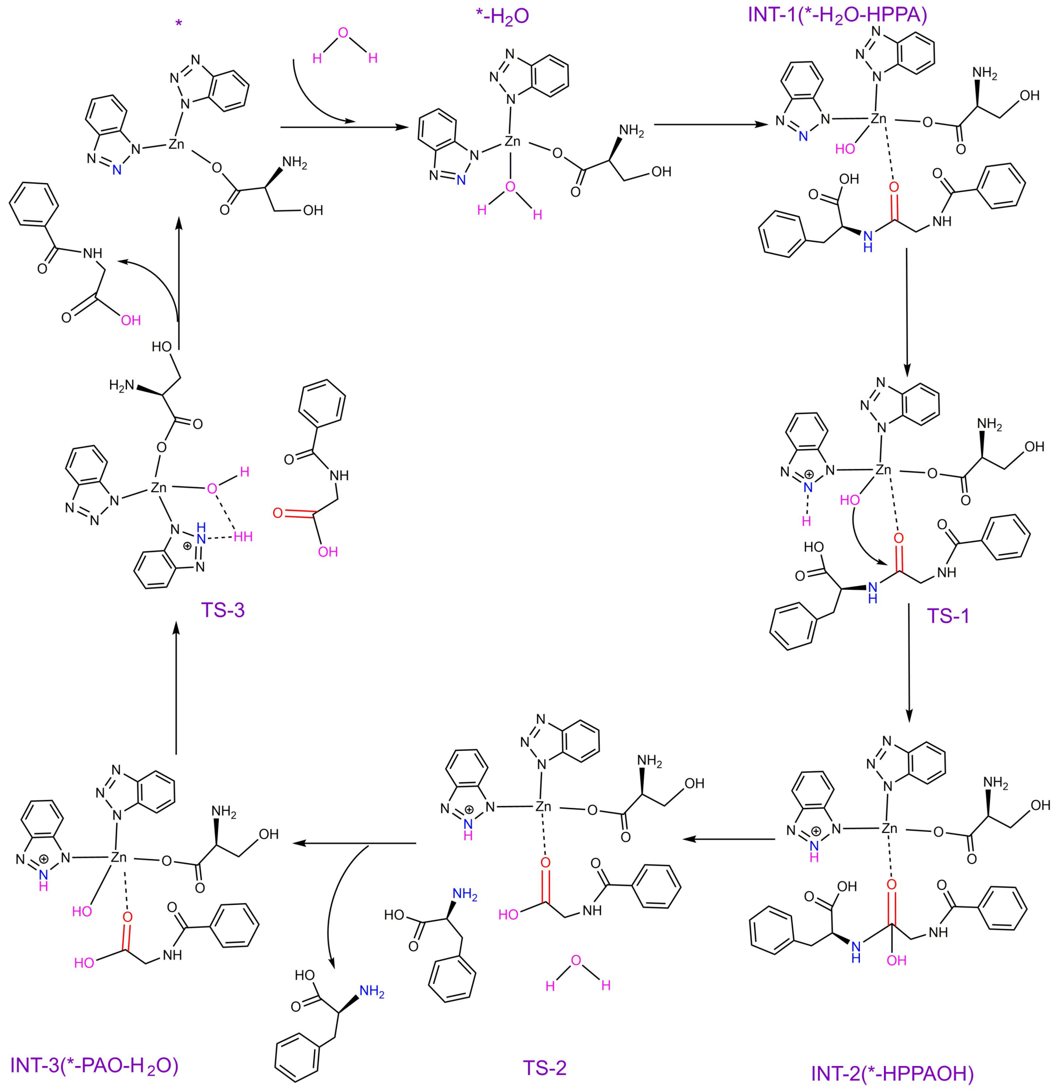

Supplementary Fig. 58. Detailed calculation path diagram of ZAF(Ser) with Lewis acid mediated active site (Zn-OH active site with serine).

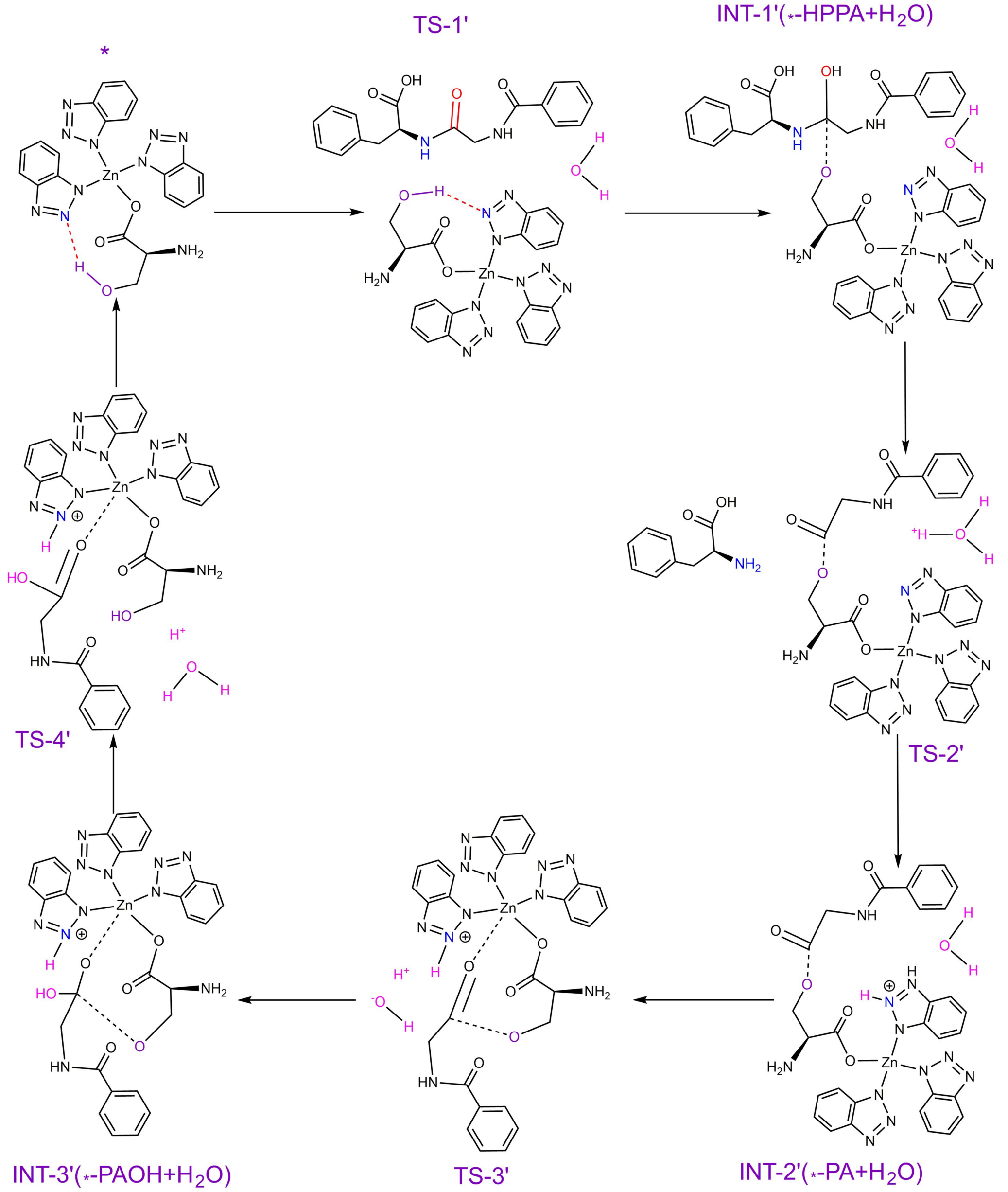

Supplementary Fig. 59. Detailed calculation path diagram of ZAF(Ser) artificial enzyme with hydrogen bonding mediated active site (Ser-O-H-N active site).

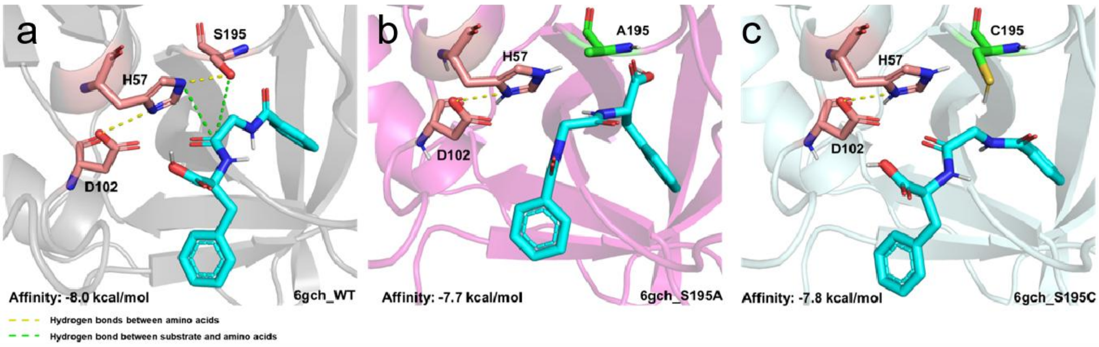

Supplementary Fig. 60. Representative conformations from MD simulations of catalytic triad in enzyme. Docking modes of the substrate hippuryl-L-phenylalanine (HPPA) to the Asp-His-Ser triad in native hdyrolase (a), Asp-His-Cys triad, with serine mutated to cysteine without hydroxyl group (b), and Asp-His-Ala triad, with serine mutated to alanine without hydroxyl group (c).

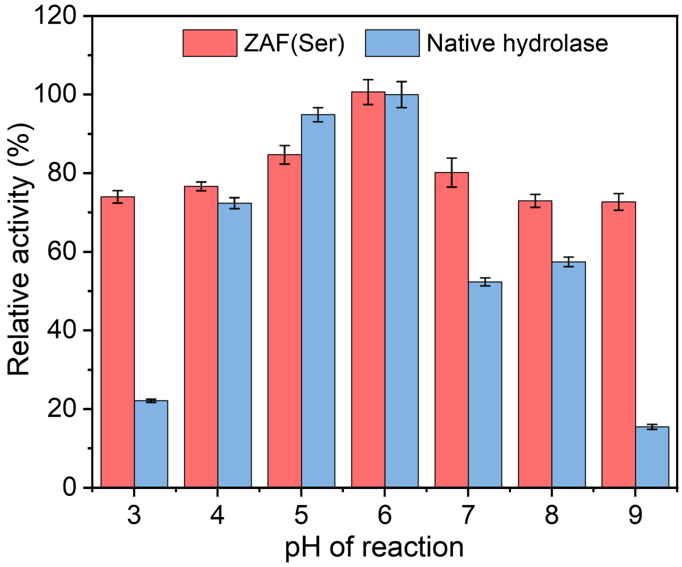

Supplementary Fig. 61. Effects of reaction pH on catalytic activity for ZAF(Ser) and native hydrolase (carboxypeptidase A). Data were represented as mean ± SD (n = 3). Source data are provided as a Source Data file.

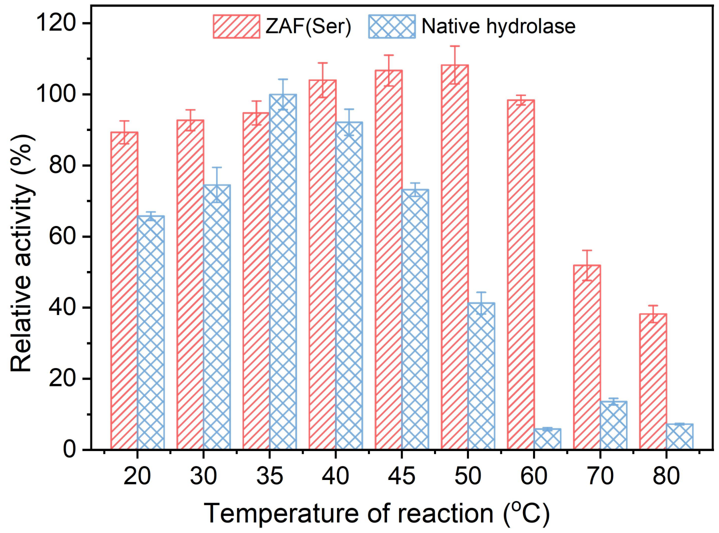

Supplementary Fig. 62. Effects of reaction temperature on catalytic activity for ZAF(Ser) and native hydrolase. Data were represented as mean ± SD (n = 3). Source data are provided as a Source Data file.

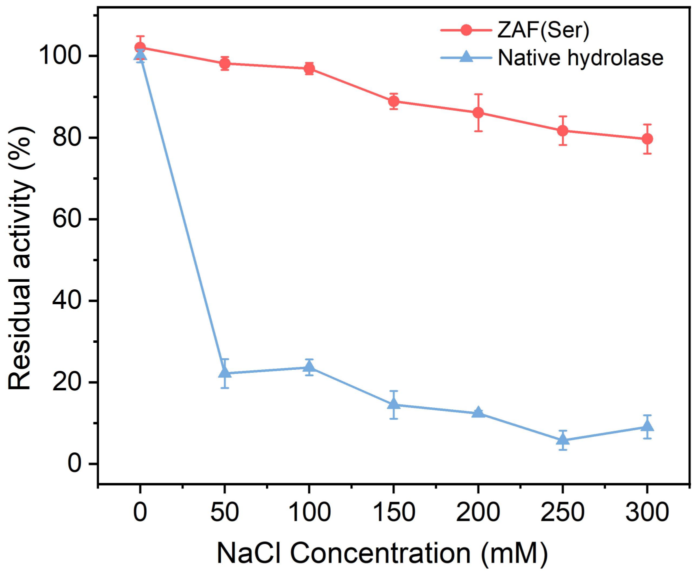

Supplementary Fig. 63. Tolerance of ZAF(Ser) or native hydrolase on varying NaCl concentration. Data were represented as mean ± SD (n = 3). Source data are provided as a Source Data file.

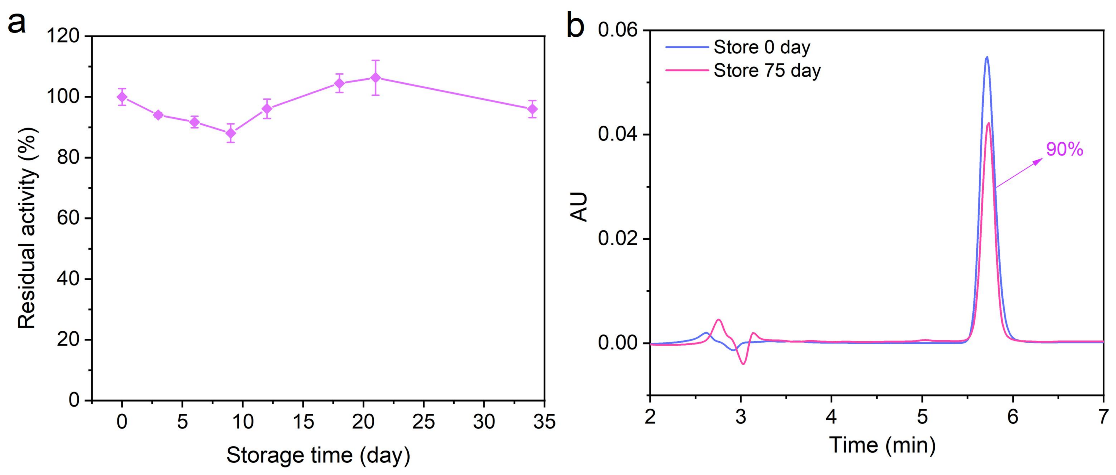

Supplementary Fig. 64. (a) Storage stability of ZAF(Ser). Data were represented as mean ± SD (n = 3). (b) HPLC diagram of the catalytic reaction of ZAF(Ser) after 75 days of storage. Source data are provided as a Source Data file.

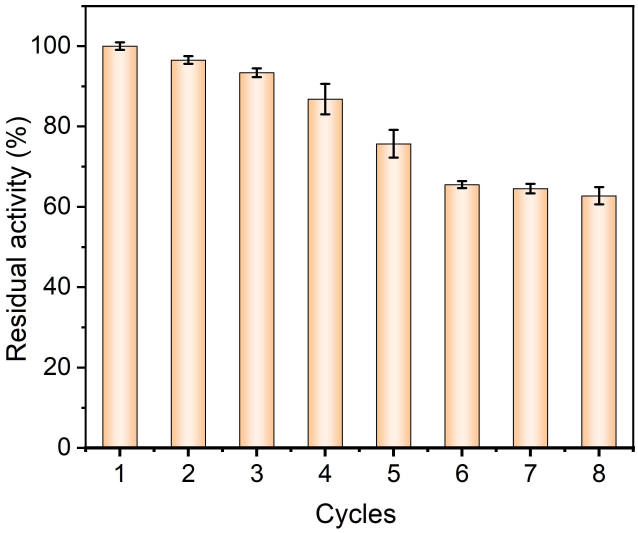

Supplementary Fig. 65. Residual activity of ZAF(Ser) in the hydrolysis reaction during the recycling and reuse process. Data were represented as mean ± SD (n = 3). Source data are provided as a Source Data file.

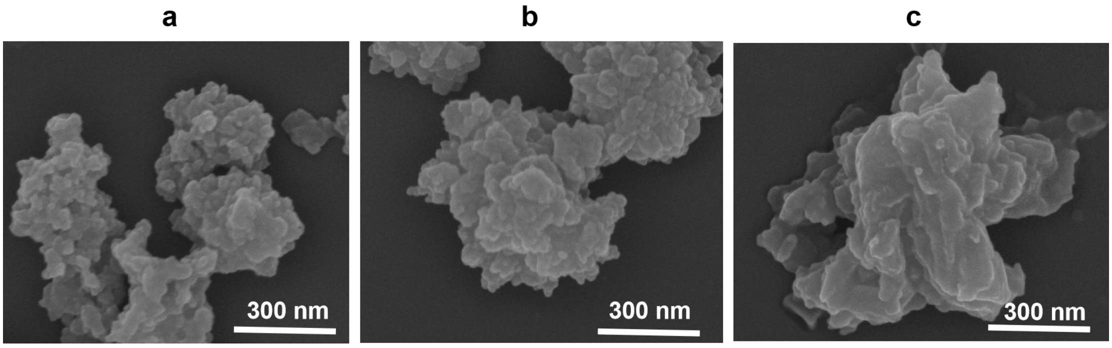

Supplementary Fig. 66. The SEM images of ZAF(Ser) after 1 cycle of reuse (a), 5 cycles (b) and 8 cycles (c) of reuse.

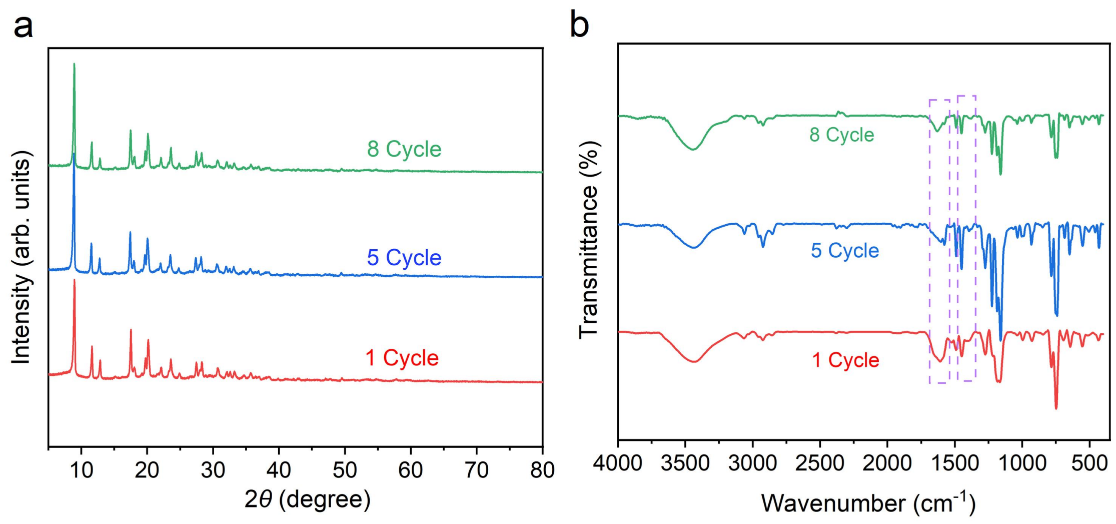

Supplementary Fig. 67. PXRD patterns (a) and FT-IR spectra (b) of ZAF(Ser) after re-use. Source data are provided as a Source Data file.

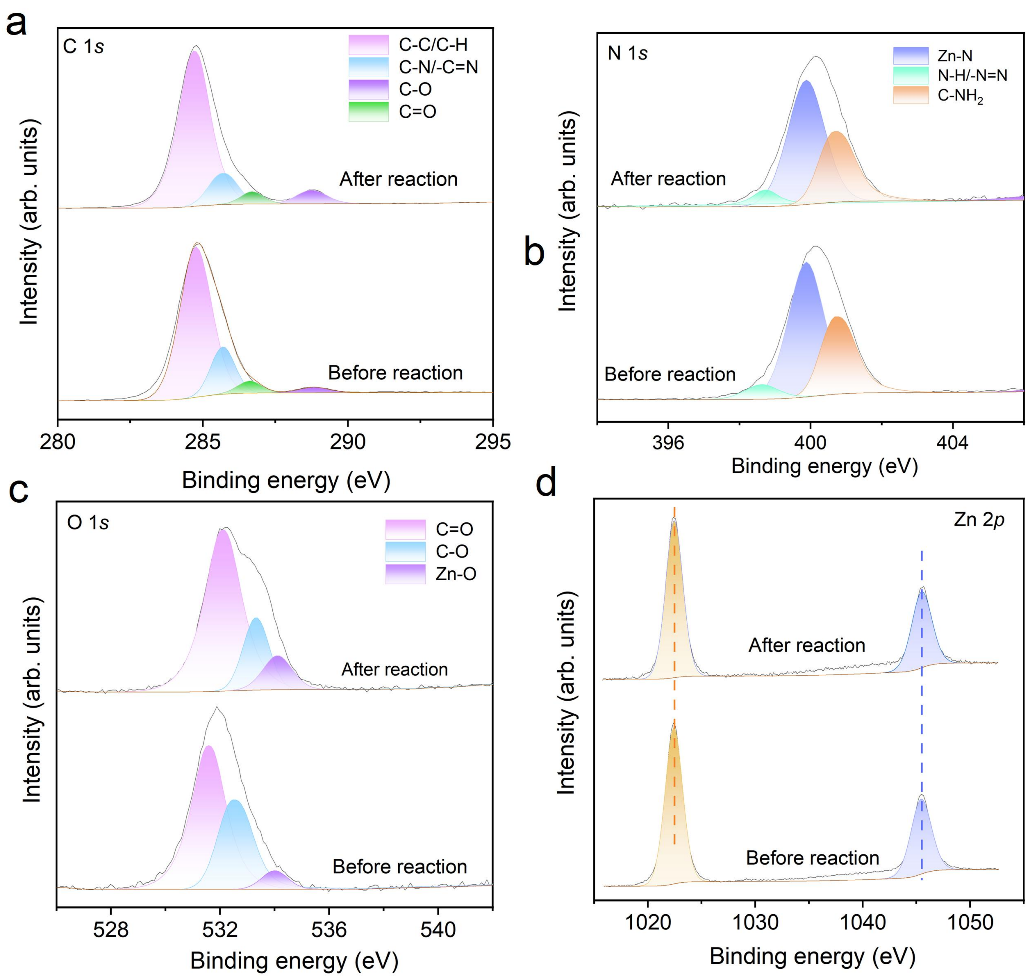

Supplementary Fig. 68. XPS spectra of ZAF(Ser) before and after reaction. High-resolution spectra of C 1*s* (a), N 1*s* (b), O 1*s* (c), and Zn 2*p* (d). Source data are provided as a Source Data file.

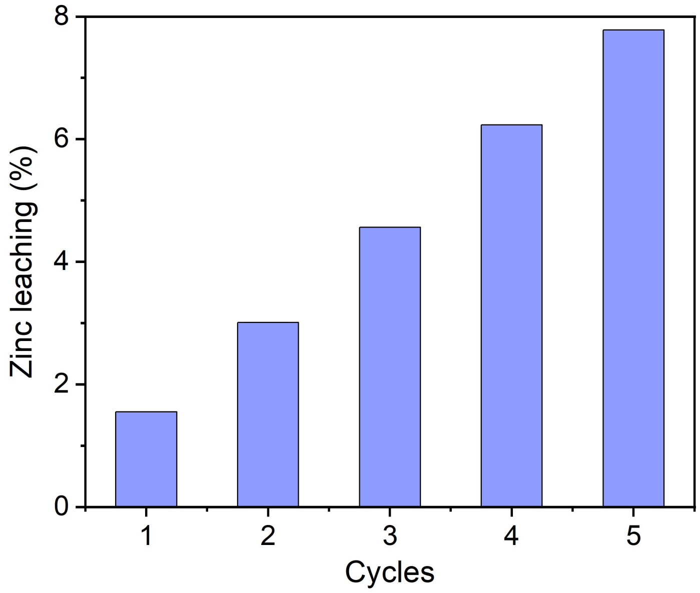

Supplementary Fig. 69. Leakage amount of Zn ion from ZAF(Ser) during re-use process determined by ICP-OES. Source data are provided as a Source Data file.

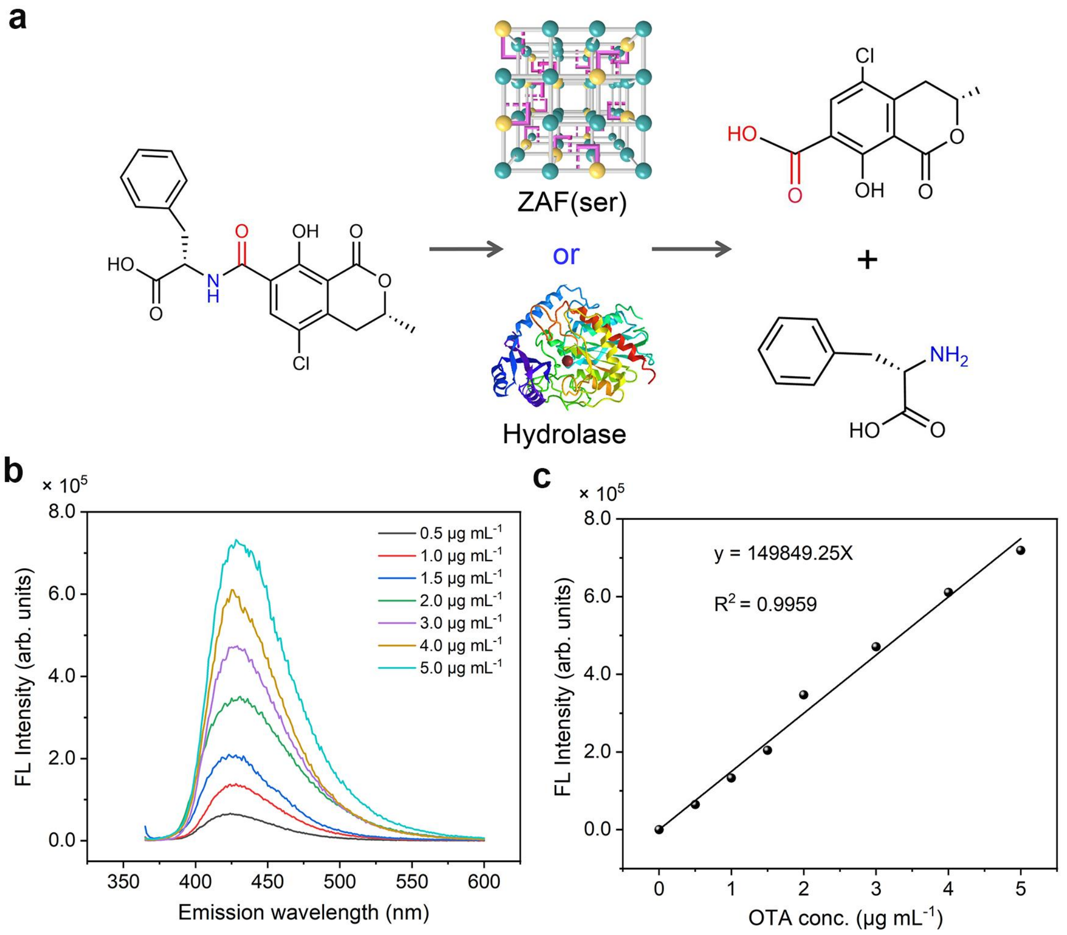

Supplementary Fig. 70. (a) Schematic diagram for catalytic degradation of ochratoxin A (OTA) catalysed by ZAF(Ser) or a native hydrolase. (b) Fluorescence spectra of OTA at different concentrations. (c) Standard curves of OTA at different concentrations. Source data are provided as a Source Data file.

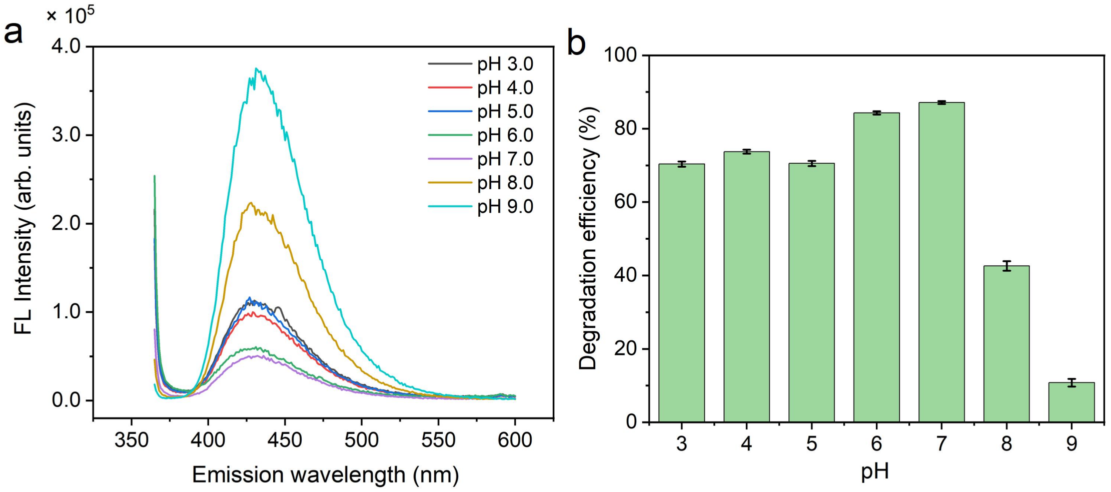

Supplementary Fig. 71. (a) Fluorescence spectra of ochratoxin A (OTA) in different reaction pH. (b) Effects of pH on degradation efficiency of OTA catalyzed by ZAF(Ser). Data were represented as mean ± SD (n = 3). Source data are provided as a Source Data file.

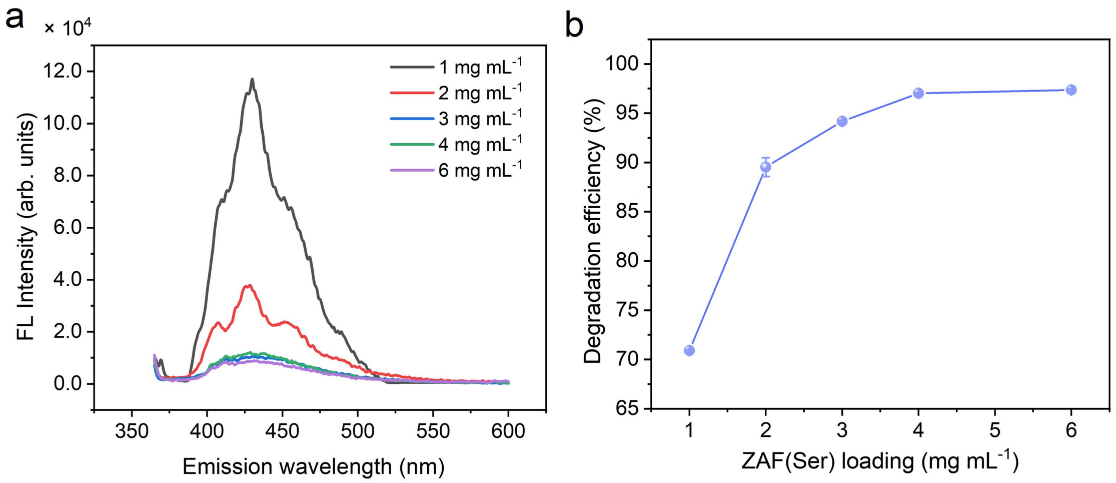

Supplementary Fig. 72. (a) Fluorescence spectra of ochratoxin A (OTA) for degradation of OTA at different ZAF(Ser) concentration and (b) the corresponding degradation efficiency of OTA. Data were represented as mean ± SD (n = 3). Source data are provided as a Source Data file.

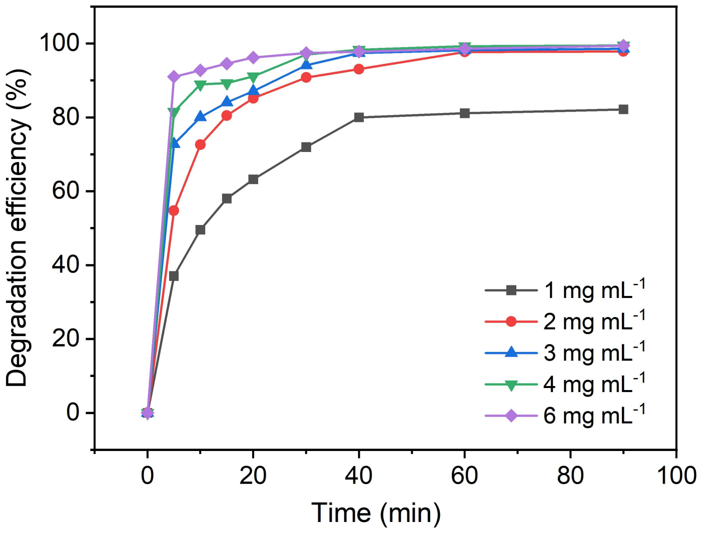

Supplementary Fig. 73. Degradation kinetics of ochratoxin A (OTA) catalyzed by different concentration of ZAF (Ser). Source data are provided as a Source Data file.

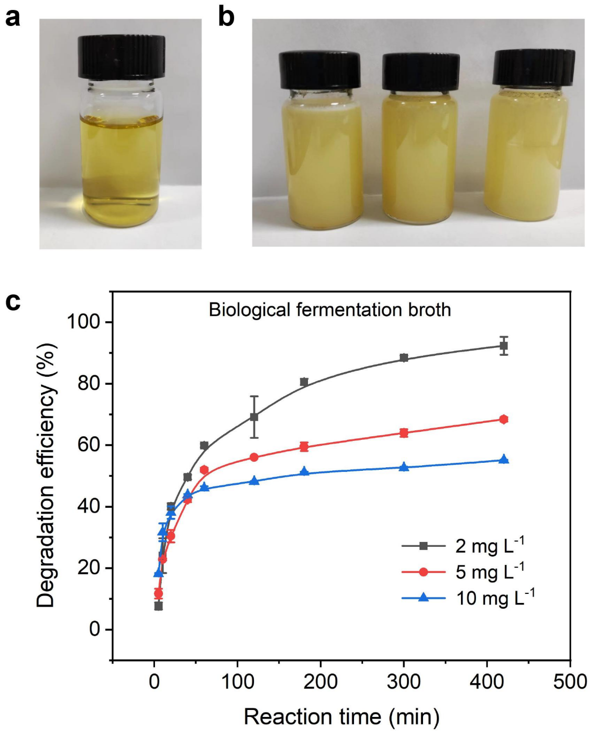

Supplementary Fig. 74. (a) The supernatant of biological fermentation broth. (b) The biological fermentation broth with addition of ZAF(Ser) artificial enzyme. (c) OTA degradation kinetics in biological fermentation broth with different OTA concentration under a fixed ZAF(Ser) concentration. Data were represented as mean ± SD (n = 3). Source data are provided as a Source Data file.

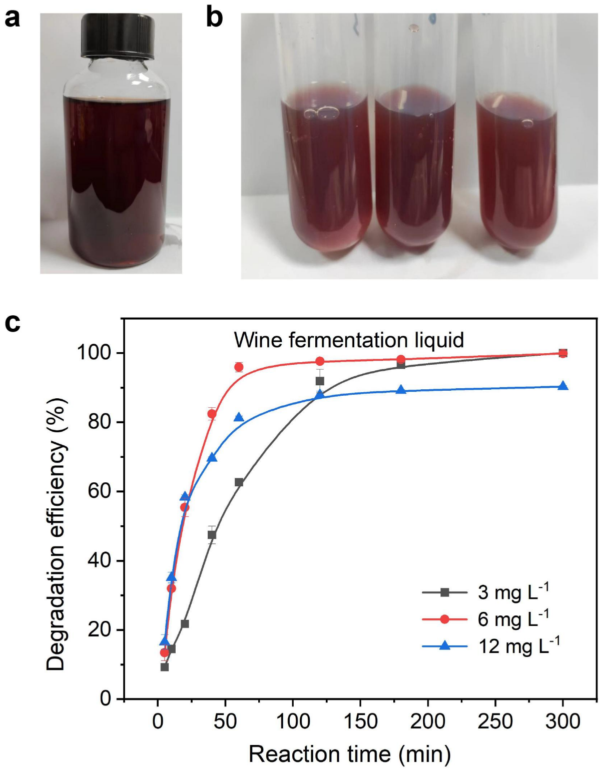

Supplementary Fig. 75. (a) Picture showing commercialized red wine from local supermarket. (b) red wine with addition of ZAF(Ser). (c) Degradation kinetic of OTA in red wine with different OTA concentration. Data were represented as mean ± SD (n = 3). Source data are provided as a Source Data file.

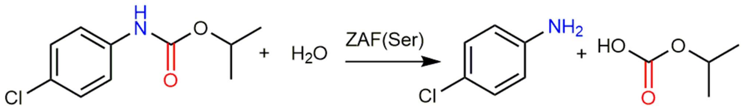

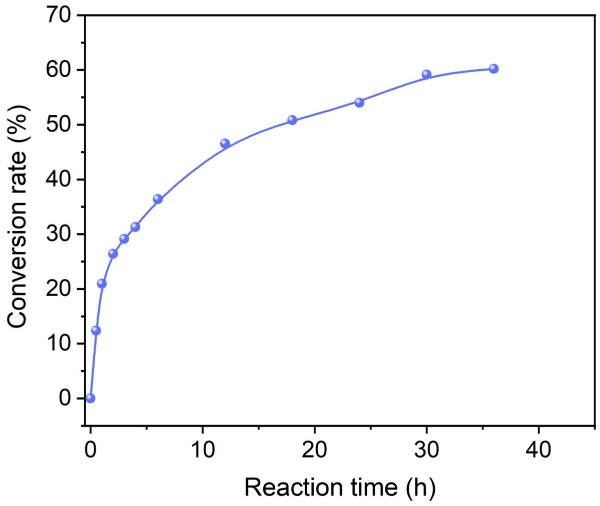

Supplementary Fig. 76. Degradation curves of chlorpropham at different reaction time catalyzed by ZAF(Ser). Data were represented as mean ± SD (n = 3). Source data are provided as a Source Data file.

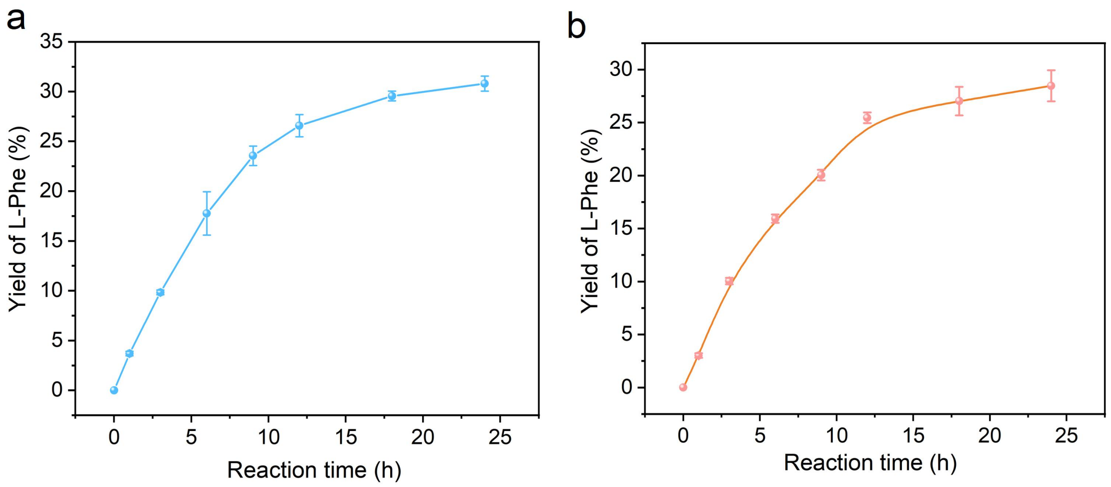

Supplementary Fig. 77. Hydrolysis kinetics of N-acetyl-L-phenylalanine (a) and N-Boc-L-phenylalanine (b) catalyzed by ZAF(Ser). Data were represented as mean ± SD (n = 3). Source data are provided as a Source Data file.

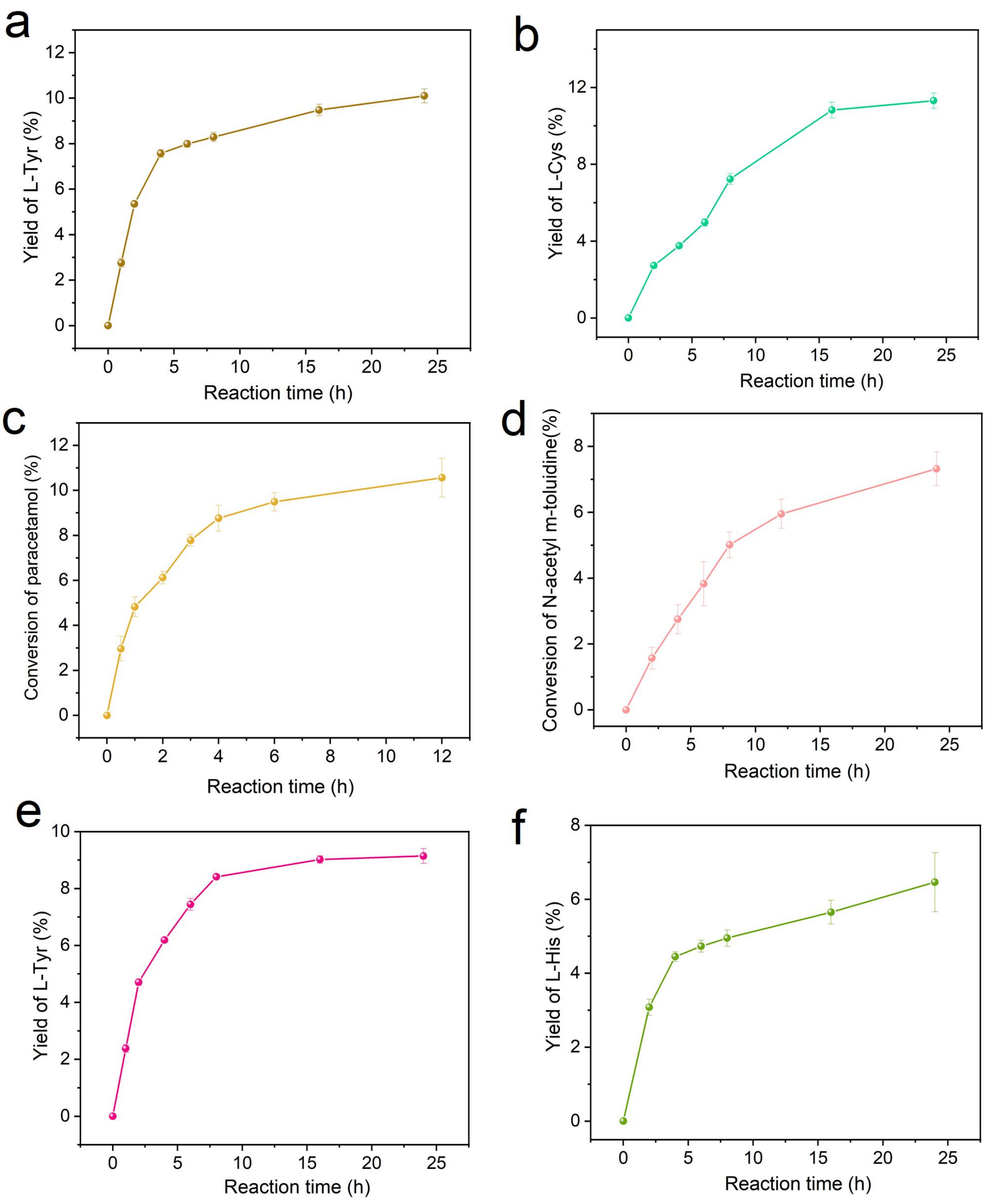

Supplementary Fig. 78. Hydrolysis kinetics of L-alanyl-L-tyrosine (a); N-acetyl-L-cysteine (b); Paracetamol (c); N-acetyl m-toluidine (d); N-acetyl-L-tyrosine (e), and N-acetyl-L-histidine (f) catalyzed by ZAF(Ser). Data were represented as mean ± SD (n = 3). Source data are provided as a Source Data file.

Supplementary Tables

Supplementary Table 1. Summary of representative hydrolytic artificial enzymes for various applications.

Enzyme-like Catalytic activity	Artificial enzymes	Application
Esterase	UiO-67	Hydrolysis of human serum albumin (HSA)[4]
Carbonic anhydrase	ZIF-8	CO_2 conversion[5]
	ZIF-100	CO_2 conversion[6]
	MFU-4l-(OH)	CO_2 separations[7]
	Zn-BTA	Trace CO_2 Capture[8, 9]
Phosphatase	Zr-MOFs	hydrolysis of Phosphate ester[10]
	UiO-66	Degradation of toxic compounds[11]
	Zr_6-MOF	Degradation of nerve agents[12]
Protease	HKUST-1	Hydrolysis of BSA and casein[13]
	MOF-808	Hydrolysis of peptide bond[14]
	UiO-66	Selective protein cleavage[15]

Supplementary Table 2. Zn content determined by ICP-OES of ZAF(Ser) obtained by varying ratio of precursors and the corresponding catalytic activities under identical amount of Zn.[a]

Molar ratio of Zn^{2+}: BTA: Ser	Zn content (wt %)	Reaction rate (μM min^{-1})	Relative activity (%)
4: 2: 2	24.4	4.0 ± 0.17	60.6
4: 2: 3	19.5	5.2 ± 0.63	78.8
4: 2: 4	22.1	4.8 ± 0.06	72.7
4: 3: 2	23.1	2.7 ± 0.12	40.9
4: 3: 3	37.3	4.6 ± 0.04	69.7
4: 3:4	28.3	5.6 ± 0.13	84.8
4: 4: 2	17.9	4.5 ± 0.24	68.2
4: 4: 3	28.3	5.7 ± 0.07	86.4
4: 4: 4	18.6	6.6 ± 0.21	100

a: Zn content (1.72×10^{-5} mol) in 6mg of ZAF is used as a reference.

Supplementary Table 3. EXAFS fitting parameters at the Zn K-edge for various samples (S_0^2=0.88).

**Sample**	**path**	**CN**	**R (Å)**	**σ^2 (Å^2)**	**ΔE_0 (eV)**	**R factor**
ZnO	Zn-O	4*	1.96 ± 0.01	0.0042	3.5 ± 0.8	0.0020
ZAF(Ser)	Zn-N(O)	3.5 ± 0.3	1.98 ± 0.01	0.0089	-1.2 ± 1.9	0.0145
	Zn-N(O)	3.0 ± 0.2	1.96 ± 0.01	0.0045		
ZAF	Zn-Zn	2.7 ± 0.4	3.22 ± 0.01	0.0072	3.9 ± 1.0	0.0119
	Zn-N(O)1	8.7 ± 1.1	3.75 ± 0.02	0.0061		

CN: coordination number for the absorber-backscatter pair. R: the average absorber-backscatter distance. σ^2: the Debye-Waller factor. ΔE_0: the inner potential correction. R factor: goodness-of-fit.

Supplementary Table 4. BET surface area, pore volume of sample, zinc content and corresponding reaction rate of different ZAF composites.[a]

Entry	Sample	Zn content (wt %)	BET surface area (m^2 g^{-1})	Total pore volume (cm^3 g^{-1}) calculated by DFT method	Reaction rate under identical amount of Zn (μM min^{-1})
0	ZAF	32.1	21.9	0.053	2.1 ± 0.04
1	ZAF(Ser)	18.6	174.8	0.256	6.6 ± 0.21
2	ZAF(Cys)	17.9	272.3	0.335	2.4 ± 0.44
3	ZAF(Ala)	19.8	99.94	0.161	1.8 ± 0.09
4	ZAF(His)	29.6	97.73	0.363	3.9 ± 0.23
5	ZAF(Glu)	20.1	73.00	0.0689	2.5 ± 0.34
6	ZAF(Asp)	29.1	62.51	0.2796	2.4 ± 0.03
7	ZAF(HSer)	31.5	97.40	0.3433	6.8 ± 0.66
8	ZAF(Thr)	29.3	23.69	0.0364	4.6 ± 0.08
9	ZAF(O-Ac-Ser)	33.3	73.51	0.3189	2.1 ± 0.03
10	ZAF(O-Btu-Ser)	29.9	67.80	0.3189	2.5 ± 0.23

a: Zn content (1.72×10^{-5} mol) in 6mg of ZAF is used as a reference.

Supplementary Table 5. BET surface area and Surface-area-normalized reaction rate of ZAF composites under identical amount of zinc.

Entry	Sample	BET surface area ($m^2\ g^{-1}$)	Surface-area-normalized reaction rate under identical amount of Zn ($g\ nM\ min^{-1}\ m^{-2}$)
1	ZAF	21.9	91.3
2	ZAF(Ser)	174.8	37.7
3	ZAF(Cys)	272.3	8.8
4	ZAF(Ala)	99.94	18.0
5	ZAF(His)	97.73	39.9
6	ZAF(Glu)	73.00	34.2
7	ZAF(Asp)	62.51	38.4

Supplementary Table 6. Elemental analysis of ZAF(Ser), ZAF(Cys), ZAF(Ala) and ZAF(HSer).

Entry	Artificial enzyme	Zn (wt %)	N (wt%)	C (wt %)	S (wt%)
1	ZAF(Ser)	18.6	28.1	47.3	-
2	ZAF(Cys)	24.9	21.5	37.3	9.9
3	ZAF(Ala)	19.8	26.6	46.1	-
4	ZAF(HSer)	31.5	22.3	40.0	-

Supplementary Table 7. Crystal data and refinement details for ZAF(Ser).

**Formula**	**ZAF(Ser)**
Moiety formula	C_{12} H_8 N_6 Zn
Formula weight	301.61
Temperature (K)	100 K
Crystal system	Monoclinic
Bond precision	C-C = 0.0030 Â
Wavelength	1.54184
a (Å)	10.00060(5)
b (Å)	12.03793(5)
c (Å)	19.87674(9)
α (º)	90
β (º)	94.3284(4)
γ (º)	90
V (Å$^{3)}$	2386.064(19)
Z	8
D_{calc} (g cm^{-3})	1.679
μ (mm^{-1})	2.820
F (000)	1216.0
N_{ref}	4804
Completeness	0.974
R(reflections)	0.0294(4789)
wR_2(reflections)	0.0719(4804)
Goodness-of-fit	1.098
N_{par}	343

Supplementary Table 8 Elemental analysis and ICP-OES of ZAF(Ser) powder and single crystal.

ZAF(Ser)	Zn (wt %)	N (wt %)	C (wt %)	Amount of Ser (wt %)	Molar ratio of Ser to Zn
**Powder**	18.6	28.1	47.3	6.1	0.2
**Single crystal**	22.2	26.6	46.5	2.7	0.091

Supplementary Table 9. The specific surface area and pore volume of x Ser@ZAF with amounts of serine doping in ZAF.[a]

Sample	Amount of added serine (mmol g^{-1} ZAF)	BET surface area (m^2 g^{-1})	Total pore volume (cm^3 g^{-1}) calculated by DFT method	Reaction rate under identical amount of Zn (μM min^{-1})
ZAF	0	21.9	0.053	2.0 ± 0.06
1 Ser@ZAF	1	11.4	0.047	2.1 ± 0.02
2 Ser@ZAF	2	10.1	0.025	2.7 ± 0.08
3 Ser@ZAF	3	7.4	0.021	3.7 ± 0.10
5 Ser@ZAF	5	5.4	0.024	5.9 ± 0.12
10 Ser@ZAF	10	6.9	0.018	7.6 ± 0.40
15 Ser@ZAF	15	3.4	0.018	8.2 ± 0.46

a: Zn content (1.72×10^{-5} mol) in 6mg of ZAF is used as a reference.

Supplementary Table 10. Elemental analysis of xSer@ZAF obtained via post-synthetic modification.

Sample	Amount of added Ser (mmol g^{-1} ZAF)	Zn (%)	N (%)	C (%)	H (%)	Amount of Ser incorporated in ZAF (mol g^{-1})	Molar ratio of Ser to Zn
ZAF	0	32.1	24.8	39.9	2.3	-	-
1 Ser@ZAF	1	31.9	20.6	35.7	2.4	1.1×10^{-3}	0.22
2 Ser@ZAF	2	31.5	20.6	36.4	2.5	1.2×10^{-3}	0.25
3 Ser@ZAF	3	29.0	21.7	38.4	2.6	1.3×10^{-3}	0.29
5 Ser@ZAF	5	24.4	22.7	40.7	2.9	1.4×10^{-3}	0.37
10 Ser@ZAF	10	21.5	23.9	43.6	3.2	1.4×10^{-3}	0.42
15 Ser@ZAF	15	19.8	25.6	45.9	3.1	1.5×10^{-3}	0.49

Supplementary Table 11. Elemental analysis of xCys@ZAF with amounts of Cys doping in ZAF.

**Sample**	**Amount of added Cys (mmol g^{-1} ZAF)**	**Zn (wt %)**	**N (wt %)**	**C (wt %)**	**S (wt %)**	**Molar ratio of Cys to Zn**
1 Cys@ZAF	1	32.8	19.9	34.2	3.0	0.19
2 Cys@ZAF	2	32.4	17.1	31.8	5.5	0.34
3 Cys@ZAF	3	32.0	15.8	29.0	7.2	0.45
4 Cys@ZAF	4	31.8	13.5	26.6	9.9	0.64
6 Cys@ZAF	6	31.6	12.6	24.0	10.2	0.70

Supplementary Table 12. Summary of different OTA detoxification methods

Method	OTA conc.	Reation condition	OTA removal efficiency
Electron beam irradiation	0.1 μg mL^{-1}	10 kGy	99.3%[16]
	1 μg mL^{-1}	16 kGy	90%[17]
		20 kGy	61%[18]
Ozone	5 μg mL^{-1}	50 mg L^{-1} ozone, 180 s	34%[19]
	17.8 μg kg^{-1}	99 mg L^{-1} ozone, 180 min	70.3%[20]
Baking	1 μg kg^{-1}	200 ℃，40 min	64%[21]
Acinetobacter calcoaceticus strain 396.1	1 μg mL^{-1}	6 d	82%[22]
Kazachstania servazzii KFGY7	1 μg mL^{-1}	2 d	88%[23]
Recombinant carboxypeptidase cp4	30 μg mL^{-1}	24 h	86.2%[24]
ZAF(Ser)	5 μg mL^{-1}	30 min	97% (This study)

Supplementary Notes

Supplementary Note 1. FT-IR spectra analysis of ZAF(Ser)

As shown in Supplementary Figure 5, the peaks at 3436, 3053 and 742-840 cm^{-1} in ZAF(Ser) correspond to the -O-H asymmetric stretching vibration, the -C-H stretching vibration and the -C-H bending vibration in the benzene ring, respectively. The FT-IR spectra of ZAF(Ser) showed the characteristic peak at 1595~1540 and 1450~1386 cm^{-1} corresponding to symmetric and asymmetric stretching vibrations of carboxyl groups, and the signals at 1278, 1226, and 1170 cm^{-1} correspond to the vibrations of -N-N- in BTA. Characteristic peak of BTA and Ser can be observed in ZAF(Ser). Moreover, the stretching vibration of Zn−N (550 cm^{-1}) and Zn-O (435 cm^{-1}) was can be observed in ZAF(Ser). These results verified the successful coordination of zinc ions with BTA and L-Ser in ZAF(Ser).

Supplementary Note 2. Investigation of residual solvent molecule inside on activity

Experiments was carried to exclude the possible effect of residual solvent molecules inside ZAF on the catalytic activity. First, deionized water (H_2O) used during the synthetic process of ZAF(Ser) was replaced with deuterium water (D_2O), which followed by washing with anhydrous methanol and drying. PXRD pattern and FT-IR spectrum (Supplementary Fig. 26) of the obtained ZAF(Ser) did not show any change. No signal peak of D_2O at 4.79 ppm in the ^1H NMR spectra of the obtained ZAF(Ser) was observed (Supplementary Fig. 27), indicating that negligible presence of D_2O inside the obtained ZAF(Ser). This ZAF(Ser) did not show activity change (Supplementary Fig. 28). Moreover, freshly prepared ZAF without washing process exhibited identical activity to the one following regular washing process, further demonstrated that even with residual H_2O inside, it did not lead to activity enhancement. Thus, the possibility of residual solvent H_2O used during the preparation of ZAF that lead to activity enhancement was excluded. Secondly, a freshly synthesized ZAF(Ser) was washed with deuterated methanol (CD_3OD) for 1 and 5 times respectively, instead of using methanol in our regular washing process and then dried overnight at 60 °C. The obtained ZAF(Ser) was acid-digested and then detected by ^1H NMR, which did not show any characteristic peak of CD_3OD at 3.31-3.34 ppm (Supplementary Fig. 29a), indicating the absence of solvent molecules inside the composite. PXRD patterns of ZAF(Ser) with CD_3OD as the washing solvent for 5 times further demonstrated the good structural integrity (Supplementary

Fig. 29 b). Activity measurement showed that both samples exhibited almost identical activity to the one washed with 3 times of methanol (Supplementary Fig. 28). Thus, the possibility that the residual solvent CH_3OH inside ZAF that lead to activity enhancement due to possible formation of CH_3OH and BTA was also excluded.

Combining the negligible presence of solvent molecules inside and also the activity measurement, the possibility that activity increase of ZAF originated from the hydrogen bonding between the ligands and the residual solvent molecules was eliminated.

Supplementary Note 3. XRD patterns of xSer@ZAF

XRD patterns in Supplementary Figure 31 demonstrated that xSer@ZAF obtained by incubating various amounts of serine with ZAF using the post-synthetic modification method retained the crystalline structure of ZAF(Ser), with peak intensity associated with ZnSer coordination weaker, confirmed the result that ZAF(Ser) and xSer@ZAF shared similar coordination micronenvironment.

Supplementary Note 4. Lewis acid mediated hydrolysis process in native metallohydrolase.

As shown in Supplementary Figure 41, enzyme binds to the substrate to form ES complexes in metallohydrolase. As the neutral water molecule was bound to the Zn^{2+} ion, the conformation changes from ES to ES*. In the second step, the zinc-bound water, activated by proton transfer to Glu, nucleophilically attack the peptide bond to form the first transition state (TS1), followed by the formation of intermediate EI1. In the third step, Glu delivers the accepted H atom to the N of peptide bond to form transition state 2 (TS2). Formation of N-H bond leads to generation of intermediate EI2. In the fourth step, the weakened C-N peptide bond breaks up and the final product is released. Simultaneously, the other H atom from H_2O molecule (now carboxylic acid proton on R_2) transfers to the anionic Glu to reach neutral and go back to starting state.

Supplementary Note 5. Lewis acid mediated hydrolysis process in ZAF and ZAF(Ser)

As shown in Supplementary Figure 43, ZAF (*) bound to the substrate to form a complex (INT-1). Zn^{2+} ion plays dual roles of polarizing both the substrate and the catalytic water molecule. In the second step, Zn-bound water molecule was activated by the Zn^{2+} ion, to generate Zn-OH^{-}, which acts as the active nucleophile and attacks the carbonyl carbon of amide bond to form a transition state 1 (TS-1) and then intermediate (INT-2). In the third step, the water molecules attack the C-N bond of the substrate to form transition state 2 (TS-2). The amine is removed from TS2 to form intermediate 3 (INT-3). Finally, the carboxylic acid-Zn dissociates to release the carboxylic acid (TS-3).

The catalytic process via the Lewis acid mediated pathway of ZAF(Ser) is similar to that of ZAF, as shown in Supplementary Figure 44. The introduction of serine into the active site results in a change in the rate-determining step. Meanwhile, the overall reaction energy barrier of ZAF(Ser) was lower than that of ZAF.

Supplementary Note 6. His-Asp-Ser Catalytic triad mediated hydrolysis process

As shown in Supplementary Figure 42, the imidazole nitrogen deprotonates the alcohol group of the serine residue, creating a powerful nucleophile for attack on the scissile amide carbonyl of the substrate in serine protease [3]. In the second step, the attack of serine on the substrate results in formation of a tetrahedral transition state 1 (TS1), which breaks down to yield acyl-enzyme intermediates, EA. In the third step, following acylation of the Ser hydroxyl, further hydrolysis of the acyl-enzyme intermediate occurs to form the second tetrahedral transition state (TS2) where water molecule acts as a nucleophile and the His imidazole acts as a general acid. Finally, hydrolysis by H_2O releases the acid product and regenerates the catalytic triad.

Supplementary Note 7. Hydrogen bond mediated hydrolysis process in ZAF(Ser)

In addition to zinc ions as the Lewis acidic active site, ZAF(Ser) polarizes the hydroxyl O through the formation of dynamic hydrogen bonds between the terminal hydroxyl and the N of the triazole group, as shown in Supplementary Figure 45. The nucleophilic O of the serine attacks the carbonyl group of the substrate, forming the transition state 1 (TS-1’). The hydroxyl oxygen of the serine binds to the carbonyl carbon of the substrate and form intermediate 1 (INT-1’). In the second step, serine further attacks the substrate, leading to the formation of the second transition state (TS-2’), and then the amine moiety is removed by hydrolysis to form intermediate 2 (INT-2’). In the third step, water molecule as nucleophile attacks the carbonyl group of ester to form transition state 3 (TS-3’). The hydroxyl group combines with the carbonyl group to form intermediate 3 (INT-3’). Removal of the hydroxyl group from INT-3' leads to the formation of TS-4’. Finally, the acid product is released by hydrolysis, and the active site of the catalyst is regenerated.

Supplementary Methods

Chemicals and materials

All chemicals and reagents were purchased from commercial sources and used without further purification. Zinc nitrate hexahydrate ($Zn(NO_3)_2 \cdot 6H_2O$, >98%), zinc acetate dihydrate ($Zn(OAc)_2 \cdot 2H_2O$, >98%), zinc chloride ($ZnCl_2$, 99%) were purchased from Sangon Biotech Co., Ltd. (Shanghai, China) . Amino acids including L-serine (Ser), L-cysteine (Cys), L-proline (Pro), L-glutamate sodium (Glu), L-alanine (Ala), L-aspartic acid (Asp), L-lysine (Lys), L-arginine (Arg), and L-histdine were purchased from Boao Biotechnology Co., Ltd. (Shanghai, China). L-amino acids are used in this study unless otherwise stated. Azole derivatives including 1,2,3-triazole (HTA), benzotriazole (BTA), hydroxybenzotriazole (HOBt), 5-methylbenzotriazole (MBTA), 5-carboxylic benzotriazole (CBTA) and benzimidazole (BDA) were obtained from Macklin Biochemical Co., Ltd., (Shanghai, China). Ochratoxin A (OTA) as standard chemical was purchased from Pribolab Co., Ltd. (Qingdao, China). Hippuryl-L-phenylalanine (HPPA, 98%) and hippuric acid (HA) purchased from Yuanye Biotechnology Co., Ltd. (Shanghai, China). Carboxypeptidase A (CPA) from bovine pancreas was purchased from Sigma-Aldrich Ltd (Darmstadt, Germany). Organic solvents of analytical purity including methanol (MeOH), ethanol (EtOH), acetone (ACE), acetonitrile (ACN), N, N-dimethylformamide (DMF), N, N-dimethylacetamide (DMAC), dichloromethane (DCM), dimethyl sulfoxide (DMSO) were purchased from Guangzhou Chemical Reagent Factory (Guangzhou, China).

Preparation of x Cys@ZAF

The xCys@ZAF with different amounts of cysteine per unit ZAF was prepared in the same way as xSer@ZAF. Only, the serine was replaced with cysteine.

Preparation of different ratio of ZAF(Cys)

$Zn(NO_3)_2 \cdot 6H_2O$ (1 mmol, 297.5 mg) and benzotriazole (1 mmol, 117.4 mg) in 30 mL of DMF were ultrasonically dissolved in a vial. Different amounts of cysteine (0.125, 0.25, 0.5, 0.75, 1 mmol) were dissolved in 10 mL of NaOH solution. The two solutions were mixed and transferred to the autoclave. The mixture was heated at 140 °C for 24 hours. After cooling to room temperature, a pale white powder was harvested by centrifugation and washed three times with DMF and methanol. Solids were dried overnight in a vacuum drying oven at 60 °C.

Preparation and analysis of ZAF(1-^{13}C Ser)

The synthesis of ZAF(1-^{13}C Ser) was identical to that of ZAF(Ser) in terms of method and procedure, except that L-serine is replaced with 1-^{13}C L-serine. 20 mg of ZAF(1-^{13}C Ser) sample was digested with 10 μL HCl and and sonicated to complete dissolution. Then, 0.8 mL of D_2O was added to dilute the sample solution. ^{13}C NMR was used to analyze the samples.

Preparation of x Ser/ZAF

Presynthesized ZAF powders (100 mg) were dispersed in 20 mL of the serine solution (2.5 mg mL^{-1}). Adsorption was carried out at 37 °C with constant shaking for 3, 6, 12, 18, 24, 36 h. At each time point, the supernatant was centrifuged from the mixture to detect the remaining serine concentration. The obtained sample was

denoted x*S*er/ZAF, where x refers to x mg of Ser initially added to 1 g of ZAF during the synthetic process.

The serine was detected by HPLC equipped with a chiral AAOA column. The composition of the mobile phase was 2 mM $CuSO_4$ solution and isopropanol with a volume ratio of 95: 5. The flow rate and detection wavelength were 0.8 mL min^{-1} and 254 nm, respectively.

Physical mixture of ZAF and ZnSer

The pseudo-ZAF(Ser) with the ratio of ZAF to ZnSer of 9: 1 was prepared based on the result of ICP-OES and elemental analysis. Then, a series of mixtures with different molar ratios (12: 1, 9: 1, 9: 2, 9: 3, 9: 4 and 9: 6) were obtained by adjusting the masses of ZAF and ZnSer. The activity assay of pseudo-ZAF(ser) was operated in the same way as that of ZAF(ser).

Physical mixture of Ser with ZAF and Ser with ZAF(Ser)

Presynthesized ZAF or ZAF(Ser) powders (6 mg) were dispersed in 1 mL reaction solution containing 3 mM HPPA. Then, 2 mL of different concentration of the serine solution (Tris-HCl, pH = 6.0) were added (3, 6, 9, 15, 30, 45 mM). The final concentration of HPPA was 1 mM. The amount of serine per unit ZAF or ZAF(Ser) in the reaction system was 1, 2, 3, 5, 10, 15 mmol g^{-1}.

XRD analysis

Powder X-ray diffraction (PXRD) patterns of obtained catalysts were recorded on a PANalytical X'pert Powder diffractometer (Netherlands) equipped with a Cu Kα radiation (λ = 0.15406 nm) at 40 kV and 40 mA with step size of 15° min^{-1}.

SEM observation.

Scanning electron microscopy (SEM) analysis was performed on a cold-field emission gun-SEM (HITACHI UHR FE-SEM SU8200) at an accelerated voltage of 10 kV. Methanol suspension of samples was dropped onto a silica wafer. After the evaporation of methanol, the silica wafer was attached to a carbon paste and then sputter-coated with gold before observation.

HAADF-STEM and EDS mapping.

Methanol suspension of samples was added on a carbon grid and dried at room temperature. High-angle annular dark-field scanning transmission electron microscopy (HAADF-STEM) images and energy-dispersive X-ray spectroscopy (EDS) mapping were conducted on JEM 2100F TEM at 200 kV.

To ensure that all O in the EDS analysis came from serine only and not H2O adsorbed from the air, we carried out experiments very carefully to avoid the long time contact of the sample with air before TEM observation and EDS mapping analysis.

For sample Storage, the freshly synthesized ZAF(Ser) were vacuum-dried overnight at 60 °C and then transferred to a sealed vacuum desiccator for storage at room temperature.

During the preparation of TEM samples, the aforementioned ZAF(Ser) powder was dispersed in anhydrous ethanol through a clean ultrasonic probe. Then, one drop containing ZAF(Ser) were placed on the carbon film and stayed for 15-20s and the excess ethanol was removed carefully using filter paper strip. After drying, the TEM

sample was quickly transferred a vacuum desiccator for temporary storage to avoid adsorption of water from the air.

During the sample transfer process, the samples were removed from the vacuum desiccator and immediately transferred to the transmission electron microscope loading tank.

FT-IR measurement.

The Fourier Transform Infrared Spectroscopy (FTIR) measurement was performed on a TENSOR27/HYPERION Fourier Transform Infrared Spectrometer. The sample powder was mixed with KBr of spectroscopic purity and pressed to disc before measurement.

Nitrogen sorption analysis

The nitrogen sorption measurement was carried out on Micromeritics Surface Area Analyser ASAP-2020 at 77 K. Samples were degassed at 120 °C for 12 h before test. The surface area was determined by Brunauer-Emmett-Teller (BET) method. The pore size distribution was analyzed by Original Density Functional Theory.

TGA and TG-DSC

TG-DSC was performed on a Netzsch STA 449F3 simultaneous thermal analyzer under N_2 atmosphere at a heating rate of 10 °C min^{-1} from 30 °C to 800 °C in an aluminum crucible. TGA was carried out on NETZSCH TG 209F1 Libra (N_2 atmosphere). The temperature range was 40 to 900 °C with the heating rate of 20 °C min^{-1}.

Nuclear Magnetic Resonance spectroscopy (^{1}H NMR) measurement

^{1}H NMR spectra were recorded on a Bruker ADVANCE 600 (600 MHz) spectrometer equipped with a 5-mm triple-resonance TCI cryogenic probe head and Z-direction pulse gradient field. D_2O-d6: δ = 4.7 ppm was used as an internal reference. Due to low solubility, 10 mg of the samples were digested with 10 drops of concentrated H_2NO_3 and sonicated until the sample was well dispersed in the acid. Then, 0.5 mL of D_2O was added to the solution.

HPLC measurement

The concentration of hippuric acid (HA) was measured on high performance liquid chromatography (HPLC) equipped with a C_{18} inverse column (Inertsil ODS-3, 250 mm× 4.6 mm i.d., 5 μm, Shimadzu, Japan). Sample solution of 10 μL was injected and eluted by using mobile phase containing 50% methanol (v/v) and 50% water (including 0.5% glacial acetic acid), with 1: 1 (v: v). The flow rate of 1.0 mL/min. The detection of HA was carried out by UV/vis absorbance at 254 nm. A standard curve of HA (0-2 mM) in the Tris-HCl buffer was pre-established.

The chlorpropham and paracetamol were detected by HPLC with mobile phase of methanol:water (80: 20, v/v). The flow rate and detection wavelength were 0.8 mL/min and 243 nm, respectively.

The products of Amino acid were analyzed through CROWNPAK®CR-I(+) column with mobile phase of aqueous solution of perchloric acid (pH = 1.5): methanol (95: 5, v/v). The detection wavelength was 220 nm.

Degradation of ochratoxin A

The degradation of ochratoxin A (OTA) was monitored by measuring the residual

amount of OTA on a Fluoromax-4P Fluorescence spectrometer. The excitation wavelength was 360 nm, and the emission spectra were recorded in the range of 350 600 nm, with the data interval of 2 nm.

ICP-OES detection and elemental analysis.

Quantification of zinc content in the samples including ZAF(Ser) and ZAF was detected via inductively coupled plasma emission spectroscopy (ICP-OES) on an iCAP 7200 (PE, USA). Elemental analysis for C, N, H in ZAF(Ser) and ZAF were detected by Thermo FLASH2000 elemental analyzer.

Single crystal X-ray crystallography.

Single crystal diffraction data of ZAF(Ser) were collected Rigaku XtalAB PRO MM007DW single crystal diffractometer at 294 K. The incident light source is Cu-Kα ray rotating-anode X-ray tube (dual wavelength). Single crystal data analysis was performed by using the shelx program. At the same time, the data and structure have been refined.

XPS analysis.

X-ray photoelectron spectroscopy (XPS) measurement was conducted on Thermo Scientific K-Alpha (USA) with a Al Kα radiation. The energy step size was 0.100 eV. The data analysis was carried out by Avantage software.

EXAFS data analysis

EXAFS fitting was performed by the Artemis module, following the EXAFS equation below:

$$\chi(k)=\sum_j \frac{N_j S_0^2 F_j(k)}{kR_j^2}\cdot\exp\left[-2k^2\sigma_j^2\right]\cdot\exp\left[\frac{-2R_j}{\lambda(k)}\right]\cdot\mathrm{Sin}\left[2kR_j+\phi_j(k)\right]$$

where S_0^2 is the amplitude reduction factor, $F_j(k)$ is the effective curved-wave backscattering amplitude, N_j is the number of neighbors in the jth atomic shell, R_j is the distance between the X-ray absorbing central atom and the atoms in the jth atomic shell, λ is the mean free path in Å, $\phi_j(k)$ is the phase shift, σ_j^2 is the Debye-Waller parameter of the jth atomic shell (variation of distances around the average R_j).

The obtained XAFS data was processed in Athena (version 0.9.26) for background, pre-edge line and post-edge line calibrations. Then Fourier transformed fitting was carried out in Artemis (version 0.9.26). The k^3 weighting, k-range of 3-12 $Å^{-1}$ and R range of 1-3 Å were used for the fitting of ZnO; k-range of 3-10.5 $Å^{-1}$ and R range of 1-2 Å were used for the fitting of samples. The four parameters, coordination number, bond length, Debye-Waller factor and E_0 shift (CN, R, ΔE_0) were fitted without anyone was fixed, the σ^2 was set.

For Wavelet Transform analysis, the $\chi(k)$ exported from Athena was imported into the Hama Fortran code. The parameters were listed as follow: R range, 1-4 Å, k range, 0-11 $Å^{-1}$ for samples; k weight, 3; and Morlet function with k=10, σ=1 was used as the mother wavelet to provide the overall distribution.

DFT calculation details

The cluster configurations of the MOF *** were extracted from the crystal structure files and then optimized under the framework of density of functional theory (DFT) with PBE0 functional and def2SVP basis set. The convergence criteria for Self-Consistent Field (SCF) is 10^{-10} Hartree for the energy change and 10^{-8} for the

maximum element of the density matrix. The default convergence criteria for optimization are to reach a maximum force of 0.00045 atomic units (a. u.) or less, reach a root mean square (RMS) gradient of 0.00030 atomic units or less, reach a maximum displacement of 0.00180 or less and reach a root mean square (RMS) displacement of 0.00120 or less. This means that the optimization will continue until the change in the gradients of all atoms in the molecule is less than 0.00045 atomic units. However, this value can be adjusted by the user depending on the specific optimization problem and desired level of accuracy. In order to describe the restraints of these ligands in our real crystals, the nitrogen atoms which are coordinated with adjacent zinc ions were frozen in our calculations.

Catalytic performance of ZAF(Ser) under varying pH

The effect of reaction pH on catalytic performance of ZAF(Ser) and native CPA was investigated by varying the reaction pH from 3 to 9, with other reaction condition remained unchanged as mentioned in the enzymatic assay. HA was dissolved in preconfigured solutions of different pH to give a final concentration of 1 mM. The activity of the native hydrolase (carboxypeptidase A, CPA) at pH 6.0 was set as 100% reference.

Recyclability of ZAF(Ser)

To evaluate the recyclability of the artificial enzyme, substrate solution (3 mL) containing1 mM HPPA (1 mM) and artificial enzyme was mixed in 50 mM Tris-HCl (50 mM, pH 6.0) at 37 °C and assayed for 1h under continuous stirring. After each cycle, the artificial enzyme was collected by centrifugation (14,000 rpm, 5 min) and

washed with DI water three times, which is then used for next cycle. The catalytic activity of artificial enzyme in the first reaction cycle was defined as 100% activity reference.

Substrate university investigation

2 mg mL^{-1} ZAF(Ser) artificial enzyme was added to the reaction flask, and then 0.3 mL of chlorpropham and paracetamol in ethanol solution (1 g mL^{-1}) and 2.7 mL of Tris-HCl buffer were injected. The mixed solution was stirred and reacted at room temperature for different time, respectively. At the end of the reaction, the solids are filtered out to obtain the sample to be tested by HPLC. N-acetyl-L-phenylalanine, N-Boc-L-phenylalanine, N-acetyl-L-cysteine, L-alanyl-L-tyrosine, N-acetyl-L-tyrosine, N-acetyl-L-histidine, and m-acetotoluidide were dissolved in Tris-HCl solution (50 mM, pH = 6) to give a final concentration of 1 mM. The above solutions were respectively added to 10 mL reaction tubes, followed by 2 mg mL^{-1} of ZAF(Ser) catalyst, set temperature for a period of time. The samples were obtained by removing the solid catalyst by filtration. Samples were analyzed by HPLC.

Supplementary References

1. Mian, M. R. et al. Insights into catalytic hydrolysis of organophosphonates at M-OH sites of azolate-based metal organic frameworks. *J. Am. Chem. Soc.* **143**, 9893-9900 (2021).

2. Nothling, M. D. et al. Synthetic catalysts inspired by hydrolytic enzymes. *ACS Catal.* **9**, 168-187 (2018).

3. Daggett, V.Schroeder, S. & Kollman, P. Catalytic pathway of serine proteases: classical and quantum mechanical calculations. *J. Am. Chem. Soc.* **113**, 8926-8935 (1991).

4. Gan, N. et al. Effects of microsize on the biocompatibility of UiO-67 from protein-adsorption behavior, hemocompatibility, and histological toxicity. *J. Hazard. Mater.* **435**, 129042 (2022).

5. Chen, J. et al. Bio-inspired nanozyme: a hydratase mimic in a zeolitic imidazolate framework. *Nanoscale* **11**, 5960-5966 (2019).

6. Jin, C. Zhang, S. Zhang, Z. & Chen, Y. Mimic carbonic anhydrase using metal–organic frameworks for CO2 capture and conversion. *Inorg. Chem.* **57**, 2169-2174 (2018).

7. Wright, A. M. et al. A structural mimic of carbonic anhydrase in a metal-organic framework. *Chem.* **4**, 2894-2901 (2018).

8. Bien, C. E. et al. Bioinspired metal–organic framework for trace CO_2 capture. *J. Am. Chem. Soc.* **140**, 12662-12666 (2018).

9. Liang, S., Wu, X. L., Zong, M. H. & Lou, W. Y. Zn-triazole coordination polymers:

Bioinspired carbonic anhydrase mimics for hydration and sequestration of CO_2. *Chem. Eng. J.* **398**, 125530 (2020).

10. Xu, M. et al. Discovery of precise pH-controlled biomimetic catalysts: defective zirconium metal-organic frameworks as alkaline phosphatase mimics. *Nanoscale* **11**, 11270-11278 (2019).

11. Lopez-Maya, E. et al. Textile/metal-organic-framework composites as self-detoxifying Filters for chemical-warfare agents. *Angew. Chem. Int. Ed.* **54**, 6790-6794 (2015).

12. Moon, S. Y. et al. Effective, facile, and selective hydrolysis of the chemical warfare agent VX using Zr6-based metal-organic frameworks. *Inorg. Chem.* **54**, 10829-10833 (2015).

13. Li, B. et al. MOFzyme: Intrinsic protease-like activity of Cu-MOF. *Sci. Rep.* **4**, 1-8 (2014).

14. Ly, H. G. T. et al. Superactivity of MOF-808 toward peptide bond hydrolysis. *J. Am. Chem. Soc.* **140**, 6325-6335 (2018).

15. Ly. H. G. T. et al. Nanozymatic Activity of UiO-66 metal-organic frameworks: tuning the nanopore environment enhances hydrolytic activity toward peptide bonds. *ACS Appl. Nano Mater.* **3**, 8931-8938 (2020).

16. Peng, C. et al. Degradation of ochratoxin A in aqueous solutions by electron beam irradiation. *J. Radioanal. Nucl. Chem.* **306**, 39-46 *(*2015).

17. Yang, K. et al. Effect of ozone and electron beam irradiation on degradation of zearalenone and ochratoxin A. *Toxins* **12**, 138 (2020).

18. Khalil, O., Hammad, A. A. & Sebaei, A. S. *Aspergillus* flavus and *Aspergillus* ochraceus inhibition and reduction of aflatoxins and ochratoxin A in maize by irradiation. *Toxicon* **198**, 111-120 (2021).

19. Krstovi, S. et al. Ozone as decontaminating agent for ground corn containing deoxynivalenol, zearalenone, and ochratoxin A. *Cereal Chem.* **98**, 135-143 (2020).

20. Vidal, A. et al. Thermal stability and kinetics of degradation of deoxynivalenol, deoxynivalenol conjugates and ochratoxin A during baking of wheat bakery products. *Food Chem.* **178**, 276-286 (2015).

21. De Bellis, P. et al. Biodegradation of ochratoxin A by bacterial strains isolated from vineyard soils. *Toxins* **7**, 5079-5093 (2015).

22. Yang, Q. Wang, J. Zhang, H. Li, C. & Zhang, X. Ochratoxin A is degraded by *Yarrowia* lipolytica and generates non-toxic degradation products. *World Mycotoxin J.* **9**, 269–278 (2016).

23. Wei, W. et al. Detoxification of ochratoxin A by *Lysobacter sp. CW239* and characteristics of a novel degrading gene carboxypeptidase cp4. *Environ. Pollut.* **258**, 113677 (2019).

24. Lai, R., Tang, W. J. & Li, H. Catalytic mechanism of amyloid-*β* peptide degradation by insulin degrading enzyme: insights from quantum mechanics and molecular mechanics style møller-plesset second order perturbation theory calculation. *J. Chem. Inf. Model.* **58**,1926-1934 (2018).

25. Wang, X. L. et al. Bottom-up synthesis of porous coordination frameworks: apical

substitution of a pentanuclear tetrahedral precursor. *Angew. Chem. Int. Ed.* **121**, 5395-5399 (2009).
