# Supplementary material for: Hydrolase mimic via second coordination sphere engineering in metal-organic frameworks for environmental remediation
Source: Nat Commun. 2023 Sep 25;14:5974. doi: 10.1038/s41467-023-41716-6 (PMC10520056; doi:10.1038/s41467-023-41716-6)
Supplement: Supplementary file 2 — Reporting Summary [file 41467_2023_41716_MOESM2_ESM.pdf]

nature portfolio

Corresponding author(s): Xiaoling Wu; Jun Ge; Wenyong Lou

Last updated by author(s): Sep 14, 2023

Reporting Summary

Nature Portfolio wishes to improve the reproducibility of the work that we publish. This form provides structure for consistency and transparency in reporting. For further information on Nature Portfolio policies, see our Editorial Policies and the Editorial Policy Checklist.

Statistics

For all statistical analyses, confirm that the following items are present in the figure legend, table legend, main text, or Methods section.

n/a	Confirmed	
☐	☒	The exact sample size (n) for each experimental group/condition, given as a discrete number and unit of measurement
☐	☒	A statement on whether measurements were taken from distinct samples or whether the same sample was measured repeatedly
☐	☒	The statistical test(s) used AND whether they are one- or two-sided<br>*Only common tests should be described solely by name; describe more complex techniques in the Methods section.*
☐	☒	A description of all covariates tested
☒	☐	A description of any assumptions or corrections, such as tests of normality and adjustment for multiple comparisons
☐	☒	A full description of the statistical parameters including central tendency (e.g. means) or other basic estimates (e.g. regression coefficient) AND variation (e.g. standard deviation) or associated estimates of uncertainty (e.g. confidence intervals)
☒	☐	For null hypothesis testing, the test statistic (e.g. F, t, r) with confidence intervals, effect sizes, degrees of freedom and P value noted<br>*Give P values as exact values whenever suitable.*
☒	☐	For Bayesian analysis, information on the choice of priors and Markov chain Monte Carlo settings
☒	☐	For hierarchical and complex designs, identification of the appropriate level for tests and full reporting of outcomes
☐	☒	Estimates of effect sizes (e.g. Cohen's d, Pearson's r), indicating how they were calculated

Our web collection on statistics for biologists contains articles on many of the points above.

Software and code

Policy information about availability of computer code

Data collection	SIESTA 4.0 version (Open source); Gaussian 16 B. 01 program
Data analysis	Origin 2021; Microsoft Office Excel 2019

For manuscripts utilizing custom algorithms or software that are central to the research but not yet described in published literature, software must be made available to editors and reviewers. We strongly encourage code deposition in a community repository (e.g. GitHub). See the Nature Portfolio guidelines for submitting code & software for further information.

Data

Policy information about availability of data

All manuscripts must include a data availability statement. This statement should provide the following information, where applicable:

- Accession codes, unique identifiers, or web links for publicly available datasets
- A description of any restrictions on data availability
- For clinical datasets or third party data, please ensure that the statement adheres to our policy

Provide your data availability statement here.

Research involving human participants, their data, or biological material

Policy information about studies with human participants or human data. See also policy information about sex, gender (identity/presentation), and sexual orientation and race, ethnicity and racism.

Reporting on sex and gender	No
Reporting on race, ethnicity, or other socially relevant groupings	No
Population characteristics	No
Recruitment	No
Ethics oversight	No

Note that full information on the approval of the study protocol must also be provided in the manuscript.

Field-specific reporting

Please select the one below that is the best fit for your research. If you are not sure, read the appropriate sections before making your selection.

☐ Life sciences ☐ Behavioural & social sciences ☒ Ecological, evolutionary & environmental sciences

For a reference copy of the document with all sections, see nature.com/documents/nr-reporting-summary-flat.pdf

Ecological, evolutionary & environmental sciences study design

All studies must disclose on these points even when the disclosure is negative.

Study description	The paper reported a metal-organic frameworks (MOFs)-based artificial enzyme system via second coordination sphere engineering to achieve high hydrolytic activity under mild conditions. This artificial hydrolase rapidly degrades highly toxic and stable fungal toxins in complex environments and achieve hydrolysis of a variety of amide compounds for environmental remediation.
Research sample	The MOF artificial hydrolase in this study was rationally designed to mimic the active center structure of carboxypeptidase, which can achieve the hydrolysis of compounds containing amide bonds. Highly toxic and stable amide compounds, such as ochratoxin, which is widely found in the food field, are prone to environmental pollution and thus hazardous to human health.<br>The hydrolytic substrates for this study, such as ochratoxin and some amide compounds, were obtained from commercially available sources.
Sampling strategy	Parallel experiments independent of each other were used in this study.
Data collection	The data were analyzed by the instrument and then the raw data were copied. The experimental data were recorded by the experimenter in a lab book and an Excel sheet, respectively.
Timing and spatial scale	According to the catalytic reaction properties, the degradation time of ochratoxin was 0-7h, and the hydrolysis time of other amide compounds was 0-36h. The reaction was sampled immediately at the end of the reaction for analysis.
Data exclusions	No data was excluded.
Reproducibility	All experimental data involving this study were conducted in three parallel experiments to reduce experimental error. For each experiment, a repeat experiment was performed to verify the reproducibility of the results.
Randomization	The MOF artificial enzyme prepared in this study was mainly applied to the degradation of highly toxic and stable toxins, and subsequently extended for the hydrolysis of some amide compounds causing environmental pollution. Randomization studies are not relevant to this study.
Blinding	As no clinical trials were involved, blinding was not relevant to this study.

Did the study involve field work? ☐ Yes ☒ No

Reporting for specific materials, systems and methods

We require information from authors about some types of materials, experimental systems and methods used in many studies. Here, indicate whether each material, system or method listed is relevant to your study. If you are not sure if a list item applies to your research, read the appropriate section before selecting a response.

Materials & experimental systems

n/a	Involved in the study
☒	☐ Antibodies
☒	☐ Eukaryotic cell lines
☒	☐ Palaeontology and archaeology
☒	☐ Animals and other organisms
☒	☐ Clinical data
☒	☐ Dual use research of concern
☒	☐ Plants

Methods

n/a	Involved in the study
☒	☐ ChIP-seq
☒	☐ Flow cytometry
☒	☐ MRI-based neuroimaging
